# Dissecting the genetic control of natural variation in sorghum photosynthetic response to drought stress

**DOI:** 10.1093/jxb/erab502

**Published:** 2021-11-16

**Authors:** Diego Ortiz, Maria G Salas-Fernandez

**Affiliations:** 1 Department of Agronomy, Iowa State University, Ames, IA 50011, USA; 2 Instituto Nacional de Tecnologia Agropecuaria, Manfredi, Cordoba 5988, Argentina; 3 University of Cambridge, UK

**Keywords:** Chlorophyll fluorescence, drought, genome-wide association study, natural variation, photosynthesis, sorghum

## Abstract

Drought stress causes crop yield losses worldwide. Sorghum is a C_4_ species tolerant to moderate drought stress, and its extensive natural variation for photosynthetic traits under water-limiting conditions can be exploited for developing cultivars with enhanced stress tolerance. The objective of this study was to discover genes/genomic regions that control the sorghum photosynthetic capacity under pre-anthesis water-limiting conditions. We performed a genome-wide association study for seven photosynthetic gas exchange and chlorophyll fluorescence traits during three periods of contrasting soil volumetric water content (VWC): control (30% VWC), drought (15% VWC), and recovery (30% VWC). Water stress was imposed with an automated irrigation system that generated a controlled dry-down period for all plants, to perform an unbiased genotypic comparison. A total of 60 genomic regions were associated with natural variation in one or more photosynthetic traits in a particular treatment or with derived variables. We identified 33 promising candidate genes with predicted functions related to stress signaling, oxidative stress protection, hormonal response to stress, and dehydration protection. Our results provide new knowledge about the natural variation and genetic control of sorghum photosynthetic response to drought with the ultimate goal of improving its adaptation and productivity under water stress scenarios.

## Teaser

Multiple genomic regions explain variation in the carbon fixation response to non-stress and drought stress in *Sorghum bicolor*, with some of these regions simultaneously controlling multiple traits.

## Introduction

Drought is a major abiotic stress that causes substantial yield losses worldwide (Li *et al.*, 2009). The large environmental variability associated with climate change may intensify future abiotic stress events (FAO, 2016), and this could lead to increased risks of food shortages, especially in regions with fast-growing populations.

Tropical C_4_ cereals such as maize (*Zea mays*) and sorghum (*Sorghum bicolor* L.) present a relative advantage in water-use efficiency over C_3_ crops in temperate regions. The anatomical and biochemical characteristics associated with the CO_2_-concentrating mechanism in C_4_ species allow plants to reach high net assimilation rates (*A*) at low stomatal conductance (*g*_s_) levels (Long, 1999; Taylor *et al.*, 2010). Sorghum can tolerate moderate drought conditions due to its deep root system that can extract water from dry soils and thus sustain stomatal opening at low water potential. Further, some sorghum accessions have leaf wax that reflects excess light and reduces cuticular conductance (Tari *et al.*, 2013). However, under a more severe water shortage, both growth and yield are reduced due to a negative effect on cell expansion, *A*, and partitioning of assimilates to harvestable organs (Peng *et al.*, 1991; Sankarapandian *et al.*, 2013).

The effects of drought on photosynthesis can be divided into stomatal and non-stomatal processes (Lawlor, 2002). As water limitation in the soil progresses, plants tend to close stomata, and thus *g*_s_ and transpiration (*E*) are reduced, limiting *A*. However, under more severe water stress, non-stomatal processes are also affected, resulting in a reduction of both leaf photochemistry and carbon metabolism, and an increase in oxidative stress (Chaves *et al.*, 2009).

A combination of drought and high light intensity generates an excess of energy that can lead to photoinhibition—the inactivation of PSII activity. Under these circumstances, photoprotection mechanisms play a key role in preventing damage to the photosynthetic machinery. Plants can dissipate excess light energy as heat through conformational changes in the light-harvesting complex (LHC). The process is mediated by the xanthophyll cycle, which consists of the de-epoxidation of violaxanthin to zeaxanthin in response to high light intensity, providing a mechanism to prevent photoinhibition and minimize oxidative stress (Demmig-Adams *et al.*, 1989; Ruban *et al.*, 2012). Changes in the efficiency of light reactions and the level of photosystem damage can be assessed using pulse amplitude modulation (PAM) fluorometers. Chlorophyll fluorescence variables have been successfully used in studies of cold, salinity, heat, and drought stresses, and the information gained can help to discover genotypic variation associated with drought tolerance (Netondo *et al.*, 2004; Hund *et al.*, 2005; Kościelniak *et al.*, 2005; Kiani *et al.*, 2008; Ortiz *et al.*, 2017).

Selection for higher photosynthetic performance has been proposed as a feasible goal for increasing crop yields (Long *et al.*, 2006; Flood *et al.*, 2011). There is extensive natural variation in *A* and the ratio between *A* and *E* (*A*:*E*) in sorghum under both non-stress and abiotic stress conditions (Kidambi *et al.*, 1990b; Peng *et al.*, 1991; Balota *et al.*, 2008; Xin *et al.*, 2009; Salas Fernandez *et al.*, 2015; Ortiz *et al.*, 2017). While variation in *A* and *A*:*E* suggests that selection for higher carbon fixation is possible, the genetic architecture of photosynthetic processes under drought stress has not been unraveled in sorghum. The objective of this study was to discover the markers/genomic regions and candidate genes associated with natural variation in gas exchange and chlorophyll fluorescence traits under well-irrigated and water-limited conditions. Our findings can be leveraged in breeding programs and exploited in engineering/editing efforts to develop superior germplasm adapted to drought-prone environments.

## Materials and methods

### Germplasm

A total of 324 accessions from the Sorghum Association Panel (SAP) that captures the worldwide natural variation in photosynthetic capacity within the species were used herein (Casa *et al.*, 2008). The SAP has been used in quantitative genetic studies to investigate numerous traits including photosynthetic response to cold stress (Sukumaran *et al.*, 2012, Morris *et al.*, 2013; Mantilla Perez *et al.*, 2014; Zhao *et al.*, 2016; Ortiz *et al.*, 2017).

### Experimental design

Accessions were evaluated in an incomplete block design, consisting of nine incomplete blocks (sets) and two replicates (biological and growth chamber variation). Each set was composed of plants representing 40 accessions and augmented with the following checks: PI656029 (B35), PI655996 (Tx430), PI533839 (Camjin), and PI564163 (BTx623). These checks were selected based on preliminary measurements of *A* under drought stress. PI564163 and PI533839 are contrasting accessions for photosynthetic capacity, while PI655996 and PI656029 are known for their drought tolerance during vegetative and reproductive stages, respectively (Sanchez *et al.*, 2002; Balota *et al.*, 2008).

### Growth conditions

In each set, 18 seeds per accession were planted in seedling trays and grown in a greenhouse for 12 d. Subsequently, two plantlets per accession were transplanted into 6 liter pots, filled with Metro Mix 900 soil (Sungro Horticulture), and one plant per accession was assigned to each of the two growth chambers equipped with metal halide and high-pressure sodium lamps (Percival, model PGW36T, capacity 11.28 m^3^). Plants were gradually adapted to high-light conditions during the first 2 weeks—from 400 μmol photons m^−2^ s^−1^ to 1000 μmol photons m^−2^ s^−1^. Growing conditions in growth chambers were 28 °C day 24 °C night, 16 h photoperiod, 1000 μmol photons m^−2^ s^−1^, and 40–60% relative humidity. Each day, light intensity was increased and decreased during the first and last 2 h of the 16 h period to simulate sunrise and sunset. Fertilization was applied as needed until the start of the water treatments with 130 ppm N of Peters Excel Cal-Mag Fertilizer (15-5-5). 

**Fig. 1.**
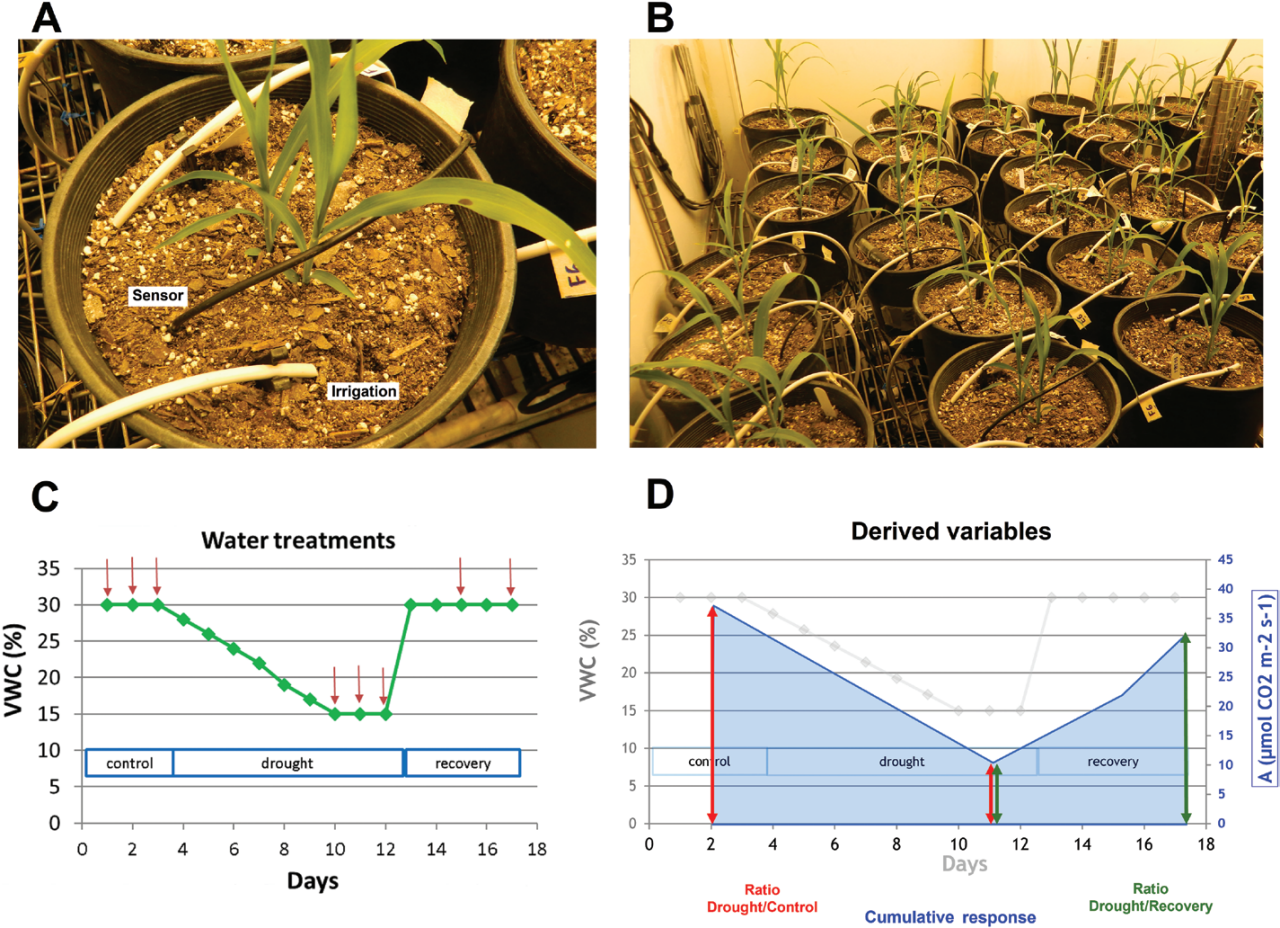
Irrigation system and treatments imposed to induce drought stress. (A) Close-up view of the irrigation and water sensor in an individual pot. (B) Arrangement of the irrigation system in growth chambers. (C) Volumetric water content (VWC) and duration of control, drought, and recovery treatments. Arrows indicate time points of photosynthesis and chlorophyll fluorescence measurements. (D) Graphical representation of the derived variables ‘cumulative response’, ‘ratio drought–control’, and ‘ratio drought–recovery’.

Soil water content was controlled in individual pots using a customized automated irrigation system described in Ortiz *et al.* (2018). Briefly, the system was based on the use of capacitance sensors in each pot, a multiplexer AM16/32B (Campbell Scientific, Logan, UT, USA), a data logger CR1000 (Campbell Scientific), and a microcontroller Mega 2560 (Arduino, Ivrea, Italy) acting as a relay driver to trigger irrigation of specific pots, if needed, to achieve the desirable volumetric water content (VWC) (Fig. 1A, B). Plants were subjected to three consecutive water treatments 30 d after planting: control or non-stress for 3 d at >30% VWC; drought that included 7 d of constant dry-down and 3 d at 15% VWC; and recovery for 5 d, when plants were re-watered and maintained at >30% VWC (Fig. 1C).

### Photosynthesis and chlorophyll fluorescence

Gas exchange and chlorophyll fluorescence measurements were collected using three LI-COR 6400 XT portable gas analyzers equipped with 6400-40 Leaf Chamber Fluorometer (LI-COR, Lincoln, NE, USA). Measurements were taken on days 1–3 of control, 8–10 of drought, and 2 and 5 of the recovery treatment (Fig. 1C). The youngest fully expanded leaf was selected for measurements during the control period, and a new leaf identified according to the same criterion was used during the drought and recovery treatments to prevent aging effects.

Dark-adapted fluorescence measurements were taken at 07.00 h. Maximum quantum yield of PSII (*F*_v_/*F*_m_) was recorded in an overnight dark-adapted leaf using an aluminum foil cover. A modulating radiation of three was used to obtain minimum chlorophyll fluorescence (*F*_0_), and a flash of 8000 μmol m^−2^ s^−1^ was applied for 3 s to record maximum chlorophyll fluorescence (*F*_m_). Results were used to calculate variable chlorophyll fluorescence (*F*_v_), as *F*_m_–*F*_0_, and maximum quantum yield of PSII as *F*_v_*/F*_m_.

After a minimum of 30 min of exposure to high-light conditions, gas exchange and light-adapted chlorophyll fluorescence parameters were measured between 09.00 h and 14.00 h in the same leaf used for dark-adapted fluorescence. Conditions in the LI-COR 6400XT leaf chamber cuvette were set to 1000 μmol photons m^−2^ s^−1^ photosynthetically active radiation (PAR), 400 ppm reference CO_2_ concentration, and 50–60% relative humidity. Leaf temperature was set to 28 °C during control and recovery days but was not controlled during the drought treatment in order to capture genotypic differences in leaf temperature regulation under stress. The fraction of blue light was 10% of the PAR level to maximize stomatal aperture. Light-adapted minimum chlorophyll fluorescence (*F*_0_ʹ) and maximum chlorophyll fluorescence (*F*_m_ʹ) were determined using a measuring light intensity of three and a saturating pulse of 8000 μmol photons m^−2^ s^−1^ for 0.8 s, respectively. Plants were allowed to stabilize for a minimum of 2 min, and subsequently four parameters were monitored for stability: *A*, *g*_s_, steady-state fluorescence, and water vapor concentration. Data were recorded when the four parameters were stable, and the overall coefficient of variation was <1.2%. The following gas exchange parameters were obtained: *A*, *g*_s_, and *E.* Chlorophyll fluorescence parameters included effective quantum yield of PSII (Φ_PSII_), efficiency of energy captured by open PSII reaction centers (*F*_v_ʹ/*F*_m_ʹ), and fraction of reaction centers that are open (qP) (Genty *et al.*, 1989; Maxwell and Johnson, 2000). Additionally, the ratio between *A* and *E* was calculated (*A*:*E*). This variable is used to detect differences in transpired water use efficiency.

Three derived variables for each photosynthetic trait were calculated to dissect the genotypic response over water treatments: (i) ‘cumulative response’ (area underneath the curve); (ii) the ratio between drought and control; and (iii) the ratio between drought and recovery (Fig. 1D). Cumulative response variables capture the overall response to the three water treatments, while ratios characterize the genotypic response to a particular treatment relative to its maximum (ratio of drought to control) or minimum (ratio of drought to recovery) values. Thus, ratio variables can provide information about the relative sensitivity of genotypes to drought and recovery.

### Statistical analysis

All variables were analyzed per water treatment using each day within a treatment as a repeated measure. Response variables were modeled using a linear mixed model and evaluated with SAS version 9.4 (SAS Institute, Cary, NC, USA). Selection of the best model was performed by comparing the Akaike information criterion (AIC) and Bayesian information criterion (BIC) of models with alternative combinations of covariates, namely leaf temperature, vapor pressure deficit, and SWC.

For *A*, *E*, *g*_*s*_, *F*_v_ʹ/*F*_m_ʹ, Φ_PSII_, and qP in control, the model was:


$$\begin{array}{l} Y_{ijklm}=\mu +\;S_{i}+\;R_{\left(i\right)j}+\;D_{k}+\;G_{l}+\;M_{m}+{\;\varepsilon \;}_{ijklm} \end{array}$$ (1)

where Y_ijklm_ is the response variable, μ is the intercept, S_i_ is the set (incomplete block) effect, R_(i)j_ is the replication nested in set effect (growth chamber), D_k_ is the day effect, G_l_ is the accession (genotypic) effect, M_m_ represents machine (gas analyzer) effects, and ε_ijklm_ is the residual.

For *A*, *E*, *g*_*s*_, *F*_v_ʹ/*F*_m_ʹ, Φ_PSII_, and qP in drought and recovery, the model was:


$$\begin{array}{l} Y_{ijklmn}=\mu +\;S_{i}+\;R_{\left(i\right)j}+\;D_{k}+\;G_{l}+\;M_{m}+\;T_{n}+{\;\varepsilon \;}_{ijklmn} \end{array}$$ (2)

where T_n_ is leaf temperature effect.

For *F*_v_/*F*_m_ in control, drought, and recovery, the model was:


$$\begin{array}{l} Y_{ijkl}=\mu +\;S_{i}+\;R_{\left(i\right)j}+\;D_{k}+\;G_{l}+{\;\varepsilon \;}_{ijkl} \end{array}$$ (3)

In all models, day, leaf temperature, and machine were considered fixed effects, while set, replication nested in set, and accession effects were considered random. For each variable, values scaled to corresponding units were obtained based on best linear unbiased predictions (BLUPs) and used as phenotypes in the genome-wide association study (GWAS). The information for descriptive statistics including estimates of correlation coefficients were generated with PROC CORR in SAS 9.4.

The intraclass correlation (*h*^2^) was estimated as:


$$\begin{array}{l} h^{2}={\sigma^{2}}_{G}/[{\sigma^{2}}_{G}+\;\;\;({\sigma^{2}}_{\;\varepsilon \;}/r)] \end{array}$$

where σ2^2^_G_ is the estimate of accession variance, σ^2^_ε_ is the estimate of error variance, and r is the number of replications. The intraclass correlation, as estimated herein, provides an estimate of repeatability.

### GWAS

A publicly available set of ~ 260 000 single nucleotide polymorphisms (SNPs) (http://www.morrislab.org/data) obtained with genotyping-by-sequencing technology (Elshire *et al.*, 2011) were utilized after filtering for <40% of missing data and minimum allele frequency of 5%. A final set of 134 200 markers were retained for the analysis.

A mixed linear model was fitted using Tassel 5.12, accounting for population structure (Q, fixed) and kinship (K, random) to minimize spurious associations (Zhang *et al.*, 2010). Q matrix, which accounts for the effects of a structured population, was estimated using STRUCTURE 2.2.3 (Pritchard *et al.*, 2000), and K matrix, which accounts for the degree of relatedness among accessions, was estimated using SPAGeDi (Hardy and Vekemans, 2002), as reported in Mantilla Perez *et al.* (2014). QVALUE software was used to calculate false discovery rates, to control for false positive associations due to multiple comparisons (Storey and Tibshirani, 2003).

### Co-localization of associated markers with known quantitative trait loci (QTL) controlling drought response or photosynthesis-related traits

QTL physical coordinates and target traits were extracted from the Sorghum QTL Atlas (Mace *et al.*, 2019) for the following 17 studies investigating drought stress response and/or photosynthesis-related traits in sorghum: (i) stay-green under field drought stress (Crasta *et al.*, 1999; Subudhi *et al.*, 2000; Tao *et al.*, 2000; Xu *et al.*, 2000; Haussmann *et al.*, 2002; Srinivas *et al.*, 2009); (ii) carbon assimilation and transpiration for pre-flowering drought applied in a greenhouse setting (Kapanigowda *et al.*, 2014); (iii) stay-green and pre-flowering tolerance under field drought conditions (Kebede *et al.*, 2001); (iv) gas exchange and/or chlorophyll fluorescence under temperature stress in controlled environments (Fiedler *et al*., 2014, 2016; Ortiz *et al.*, 2017); (v) grain yield, morphology, and plant architecture under pre-flowering drought conditions in field experiments (Phuong *et al.*, 2013); (vi) chlorophyll content and biomass under cold and heat stress (Chopra *et al.*, 2017); (vii) stay-green and grain yield under field drought conditions (Sabadin *et al.*, 2012; Rama Reddy *et al.*, 2014); (viii) leaf drying rate under drought imposed in a greenhouse setting (Sakhi *et al.*, 2013); and (ix) stay-green, agronomic traits, chlorophyll content, and chlorophyll fluorescence under drought in field trials (Sukumaran *et al.*, 2016). The co-localization of significant markers with these published QTL was investigated using the Sorghum genome v3 physical coordinates.

### Identification of candidate genes in significantly associated regions

Linkage disequilibrium (LD) regions were defined as in Ortiz *et al.* (2017). Candidate genes within 55 kb from significant markers were identified using the Sorghum genome v3. Of this comprehensive list, the most promising candidates were selected based on the following criteria: (i) their expression in leaves according to 48 microarray and RNAseq experiments (McCormick *et al.*, 2018); (ii) their differential expression under pre-flowering drought conditions based on a transcriptome field study of elite inbred lines BTx642 (post-flowering drought tolerant) and RTx430 (pre-flowering drought tolerant) (Varoquaux *et al.*, 2019); and/or (iii) predicted peptide signal targeting the encoded protein to the chloroplast or mitochondria according to TargetP-2.0 (Almagro Armenteros *et al.*, 2019). This reduced list of suggested genes for future validation studies was further reduced based on their predicted functions and homology to genes with a potential role in drought stress response and carbon fixation.

## Results

### Analysis of phenotypes

Our automated irrigation system provided the ability to control the dry-down of potted plants during a 7 d period and was able to maintain a final VWC of 15±5% (Supplementary Table S1). This precise soil water control is comparable with those attained with large-scale automated phenotyping platforms (Granier *et al.*, 2006; Finkel, 2009; Junker *et al.*, 2015).

**Fig. 2.**
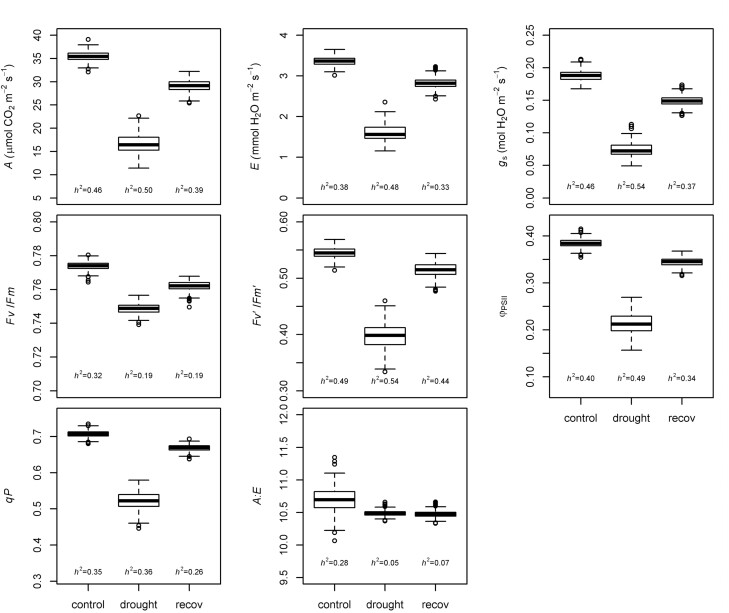
Box plots of gas exchange and chlorophyll fluorescence traits based on BLUPs for control, drought, and recovery periods. *h*^2^, heritability; *A*, photosynthesis; *E*, transpiration, *g*_s_, stomatal conductance, *F*_v_/*F*_m_, maximum quantum yield of PSII, Φ_PSII_, effective quantum yield of PSII; *F*_v_ʹ/*F*_m_ʹ, efficiency of energy captured by open PSII reaction centers; qP, photochemical quenching or fraction of PSII reaction centers that are open, and *A*:*E*, ratio between photosynthesis and transpiration.

Water treatments affected all photosynthetic parameters, as expected, with the largest among-treatment differences observed between control and drought, followed by differences between control and recovery (Fig. 2). In general, relative differences attributed to drought were greater for gas exchange variables (52–61%) than for chlorophyll fluorescence traits (3–44%). Stomatal conductance was the most drastically reduced parameter during the drought period in all genotypes, while *F*_v_/*F*_m_ and *A*:*E* had the minimum response to stress (2% reduction).

There were multiple significant estimates of correlation among traits within treatments (Supplementary Tables S2–S4). Gas exchange variables were highly correlated with each other, and *A* was consistently correlated with Φ_PSII_ in all water treatments (Supplementary Table S2; Supplementary Figs S1, S2). Even though qP and *F*_v_ʹ/*F*_m_ʹ were both highly correlated with Φ_PSII_, the correlation between the two former variables was low to intermediate within water treatments (*r*=0.19–0.61) (Supplementary Tables S2–S4). In general, *F*_v_/*F*_m_ presented low or no correlation with most variables under non-stress (Supplementary Table S2), but those associations increased under drought (Supplementary Tables S3, S4).

The ANOVA confirmed that genotypes from the SAP exhibit significant variation in all traits and water treatments, except for *A*:*E* during drought and recovery periods (Supplementary Table S5). Among the fixed effect factors, T_leaf_ was significant for most variables except *g*_s_ in recovery, while machine was not significant for *F*_v_ʹ/*F*_m_ʹ during the same period. In general, estimates of repeatability were intermediate (0.26–0.54), but low for *F*_v_/*F*_m_ and *A*:*E* during the drought and recovery periods, ranging from 0.05 to 0.19 (Fig. 2).

### Genome-wide association results

**Fig. 3.**
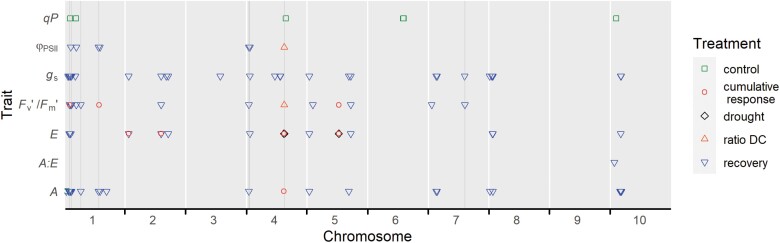
Summary of genome-wide associations for photosynthesis and chlorophyll fluorescence using 324 diverse sorghum accessions. Only significant SNPs/regions are represented by a dot. *A*, photosynthesis (μmol CO_2_ m^–2^ s^–1^); *E*, transpiration rate (mmol H_2_O m^–2^ s^–1^); *g*_s_, stomatal conductance (mol H_2_O m^–2^ s^–1^); *F*_v_ʹ/*F*_m_ʹ, efficiency of energy captured by open PSII reaction centers; Φ_PSII_, effective quantum yield of PSII; qP, photochemical quenching or fraction of PSII reaction centers that are open; and *A*:*E* ratio between photosynthesis and transpiration. Vertical blue lines highlight genomic regions associated with multiple traits and/or treatments.

Sixty regions defined by LD (labeled as chromosome#_region#) were significantly associated with seven photosynthetic traits throughout three water treatments and derived variables (Table 1; Supplementary Table S6; Supplementary Figs S3–S10). Of the total 181 SNP–trait correlations, the majority (88%) corresponded to the recovery treatment, while only small percetanges occurred during drought, control (non-stress), or as a cumulative response (Table 1; Fig. 3). Even though there were no markers consistently associated with the same variable in different water treatments, there were cases in which regions in LD were significant for a particular trait–treatment, and its derived variable. For example, *S5_42764230* was significant for *E* control and *E* cumulative response. GWA signals were detected in all chromosomes except chromosome 9 and included a variable number of SNPs (1–31) for a single trait (Fig. 3). The percentage of phenotypic variation explained by a marker ranged from 5% to 12% (Table 1). The associated LD regions harbor a total of 796 genes located within 55 kb from the significant SNPs (Supplementary Table S7). This list includes genes with predicted functions related to membrane transporters, transcription factors, oxidative stress, protein kinases, and peroxidases, among others (Supplementary Table S7, S8).

**Table 1.** Summary of regions significantly associated with variation in photosynthesis and chlorophyll fluorescence traits during three water treatments

Trait	FDR threshold^a^	Corresponding*P*-value^b^	Chromosome	*R* ^2^ range	No. of significant LD regions
*A* control	0.073	5.62E-07	1	0.09–0.09	1
*A* recovery	0.132	5.64E-05	1,4,5,7,8,10	(0.051–0.104)	24
*A* cumulative response	0.05	4.50E-07	4	0.09–0.09	1
*E* drought	0.155	5.36E-06	4,5	0.077–0.111	3
*E* recovery	0.13	2.03E-05	1,2,4,5,8,10	0.06–0.115	12
*E* cumulative response	0.119	5.32E-06	2,4,5	0.077–0.105	5
*g*_s_ recovery	0.087	4.12E-05	1,2,3,4,5,7,8,10	0.055–0.128	31
*F*_v_ʹ/*F*_m_ʹ recovery	0.059	9.45E-06	1,2,4,5,7	0.064–0.128	10
*F*_v_ʹ/*F*_m_ʹ ratio DC	0.111	9.36E-07	4	0.11–0.11	1
*F*_v_ʹ/*F*_m_ʹ cumulative response	0.184	4.98E-06	1,5	0.074–0.095	3
Φ_PSII_ ratio DC	0.157	1.28E-06	4	0.108–0.108	1
Φ_PSII_ recovery	0.127	1.95E-035	1,4	0.059–0.104	6
qP control	0.177	1.30E-05	1,4,6,10	0.066–0.089	7
*A*:*E* recovery	0.116	1.73E-06	10	0.08–0.08	1

DC, drought–control.

*P-*value corresponds to significance level of the region–trait association.

False discovery rate (FDR) threshold for each trait in the GWAS. FDR was calculated, according to Storey and Tibshirani (2003), to control for false-positive associations due to multiple comparisons.

**Fig. 4.**
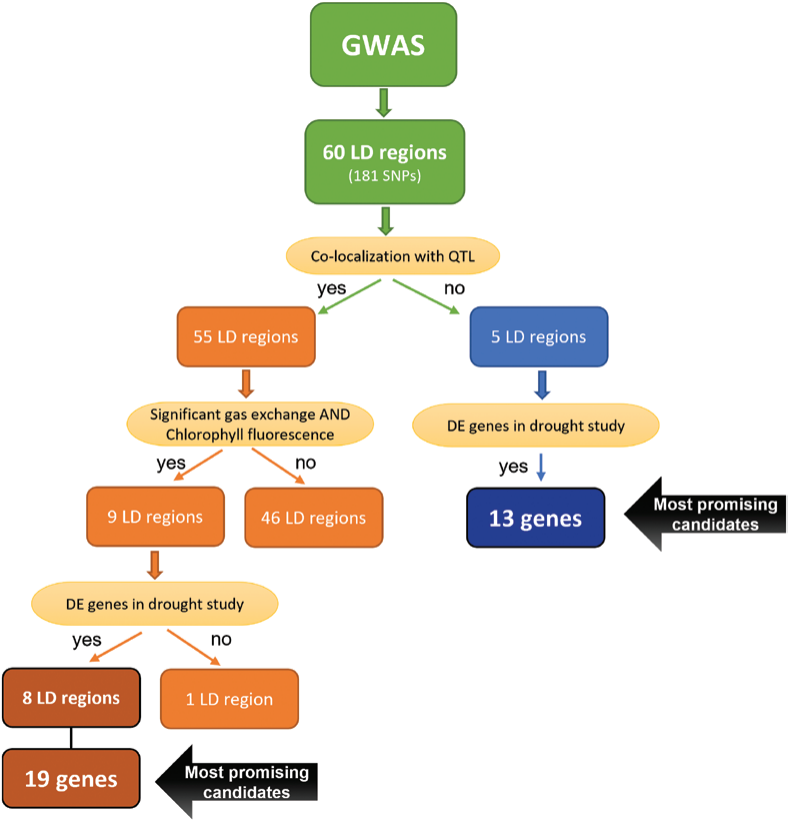
Flow chart of comparative analyses conducted to identify the most promising candidate genes within significantly associated LD regions. DE, differentially expressed genes based on RNAseq study conducted under drought stress by Varoquaux *et al.* (2019).

To prioritize discoveries, regions were initially grouped based on their co-localization with previously reported QTL controlling either diverse yield/agronomic characteristics under drought or photosynthesis-related traits. Out of the 60 significant LD regions, 55 overlapped with known QTL, providing independent support for the newly characterized natural variation in sorghum photosynthetic capacity (Fig. 4; Table 2; Supplementary Table S8). The remaining five regions represent novel loci that contain 13 genes differentially expressed under pre-flowering drought stress (Fig. 4; Table 3; Supplementary Table S7, S8) (Varoquaux *et al.*, 2019). The coincident association with gas exchange and chlorophyll fluorescence parameters was the subsequent criterion used to select nine out of the 55 regions. Those nine chromosomal intervals harbor 173 predicted genes whose expression levels in response to pre-flowering drought were extracted from the RNAseq study conducted by Varoquaux *et al.* (2019). Of the 57 differentially expressed genes (Supplementary Table S9), 20 had predicted functions related to drought stress response or carbon fixation based on homology with Arabidopsis, maize, or rice genes, and 10 of them had a predicted chloroplast or mitochodria targeting sequence as determined by TargetP (Table 2).

**Table 2.** Subset of significant genome-wide associations for both gas exchange and chlorophyll fluorescence traits that co-localized with known QTL for agronomic traits under drought- or photosynthesis-related parameters

GWAS					Overlapping QTL^a^		Promising candidate genes			
Region^b^	Physical interval^c^	No. of SNPs	No. of genes^d^	Traits	QTL ID	Traits	ID	Predicted function	Signal^e^	DE drought^f^
**1_8**	7959919	1	12	*A* recov, *g*_s_ recov, *F*_v_ʹ/*F*_m_ʹ recov	QCHLC1.27, QCHLC1.3 QDMGR1.22 QGLFA1.1	Chlorophyll content Dry matter growth rate Green leaf area	Sobic.001G103300	ER-type calcium-transporting ATPase		Tx430
**1_15**	9983041–11205226	5	34	*A* recov, *g*_s_ recov, *E* recov, *F*_v_ʹ/*F*_m_ʹ recov, ΦPSII recov	QCHLC1.27, QCHLC1.3 QCO2A1.1 QDMGR1.2, QDMGR1.22 QGLFA1.4 QTNGL1.2, QTNGL1.3	Chlorophyll content CO_2_ assimilation ratio Dry matter growth rate Green leaf area Total no. of green leaves	Sobic.001G126500 Sobic.001G127500 Sobic.001G128600 Sobic.001G129700 Sobic.001G140400 Sobic.001G140700	RNA helicase Chaperone ABC1 prot kinase Unknown function germin-like protein 4 EPF-like Pyruvate kinase family protein	Mt Chl Chl	Tx642 Both Tx642 Both Tx642 Tx430
**1_19**	26573871	1	6	*A* recov, *F*_v_ʹ/*F*_m_ʹ recov	QCHLF1.31 QDMGR1.22 QGLFA1.5, QGLFA1.6 QLFTE1.1, QLFTE1.2 QPSII1.42, QPSII1.43, QPSII1.44 QSTCD1.24 QTRSP1.2 QWUEF1.1	Chlorophyll fluorescence Dry matter growth rate Green leaf area Transpiration rate Effective quantum yield PSII stomatal conductance Transpiration Water use efficiency	Sobic.001G248300 Sobic.001G248500	Acylamino acid-peptidase-related glutathione reductase	Chl Chl	Tx430 Tx430
**1_20**	56058472	1	6	*A* recov, ΦPSII recov	QDMGR1.23 QGLFA1.10, QGLFA1.9 QSTCD1.25	Dry matter growth rate Green leaf area stomatal conductance	Sobic.001G286100	Inositol 1,3,4-trisphosphate 5/6-kinase family protein		Both
**4_1**	3797939–3800779	3	20	*A* recov, ΦPSII recov	QCHLF4.18 QDMGR4.9 QGYLD4.8 QTNGL4.1, QTNGL4.2	Chlorophyll fluorescence Dry matter growth rate Grain yield Total no. of green leaves	Sobic.004G046200 Sobic.004G046500	Sulfotransferase 4A Small GTP-binding protein	Chl	Tx642 Both
**4_2**	3813276–5677473	7	56	*E* recov, *g*_s_ recov, *F*_v_ʹ/*F*_m_ʹ recov, ΦPSII recov	QCHLF4.18 QDMGR4.9 QGYLD4.8 QTNGL4.1, QTNGL4.2	Chlorophyll fluorescence Dry matter growth rate Grain yield Total no of green leaves	Sobic.004G047100 Sobic.004G069850 Sobic.004G070000 Sobic.004G070200	EPS15 homology domain 1 Cyclase family protein Cyclase family protein RNA-metabolizing metallo-beta-lactamase	Chl/mt Chl	TX642 Tx642 Both Both
**4_7**	62405510–62425357	2	23	*E* drought, E cum res, *F*_v_ʹ/*F*_m_ʹ ratio DC, ΦPSII ratio DC	QCHLF4.16, CHLF4.17	Chlorophyll fluorescence	Sobic.004G281600 Sobic.004G282000 Sobic.004G282400	Mechanosensitive ion channel Pentatricopeptide repeat prot Rho GTPase activating prot	Mt Chl	Both Both Both
**7_3**	60259969	2	14	*g*_s_ recov, *F*_v_ʹ/*F*_m_ʹ recov	QTRSP7.1	Transpiration	Sobic.007G168000	Chlorophyllase 1		Both

Promising candidate genes were selected based on differential expression under drought conditions in a previous study, predicted peptide signal targeting to chloroplast or mitochondria, and predicted function.

QTL physical coordinates and target traits were extracted from the Sorghum QTL Atlas (Mace *et al.*, 2019) for the following 17 studies investigating drought stress response and/or photosynthesis-related traits in sorghum: Crasta *et al.*, 1999; Haussman *et al.*, 2002; Srinivas *et al.*, 2009; Subudhi *et al.*, 2000; Tao *et al.*, 2000; Kapanigowda *et al.*, 2014; Kebede *et al.*, 2001; Fiedler et al., 2014, 2016; Ortiz *et al.*, 2017; Phuong *et al.*, 2013; Chopra *et al.*, 2017; Rama Reddy *et al.*, 2014; Sabadin *et al.*, 2012; Sakhi *et al.*, 2013; Sukumaran *et al.*, 2016; Xu *et al.*, 2000.

Significantly associated genomic regions are labeled as chromosome#_region#.

Physical position based on sorghum genome v.3.

Total number of predicted genes within 55 kb of significant markers according to sorghum genome v.3.

Predicted N-terminal transit peptide targeting protein to mitochondria or chloroplast, according to TargetP-2.0 (Almagro Armenteros *et al.*, 2019).

Indicates if differentially expressed under pre-flowering drought stress in Tx642, Tx430, or both lines, according to Varoquaux *et al.* (2019).

**Table 3.** Significant genome-wide associations that do not co-localize with any known QTL

GWAS					Candidate genes			
Region^a^	Physical interval^b^	No. of SNPs	No. of genesc^b^	Traits	Most promising	Predicted function	Signal^d^	DE drought^e^
**1_22**	58204300–58204403	4	11	*A* recov, ΦPSII recov	Sobic.001G300201 Sobic.001G300700	HLH transcription factor Expansin B		Tx430 Tx642
**1_23**	58515970	1	8	*A* recov	Sobic.001G302300	Unknown function		both
**2_3**	58959838	1	7	*E* recov, *g*_s_ recov, *E* cum res, *F*_v_ʹ/*F*_m_ʹ recov	Sobic.002G199900 Sobic.002G200200	Unknown function CAAX-Nterminal protease	Chl	Both Tx642
**5_4**	68758327- 68758354	2	11	*A* recov, gs recov	Sobic.005G202600	Tropinone reductase		Tx430
**5_5**	71668462- 71668533	3	20	*E* recov, *g*_s_ recov, *F*_v_ʹ/*F*_m_ʹ recov	Sobic.005G229000 Sobic.005G229400 Sobic.005G229500 Sobic.005G229600 Sobic.005G229700 Sobic.005G229900 Sobic.005G230100	SC35-like splicing factor 30 GRAS family TF GRAS family TF GRAS family TF GRAS family TF GRAS family TF GRAS family TF		Tx642 Tx642 Tx642 Tx642 Tx642 Tx430 Tx642

Promising candidate genes were selected based on differential expression under drought conditions in a previous study, predicted peptide signal targeting to chloroplast or mitochondria, and predicted function.

Significantly associated genomic regions are labeled as chromosome#_region#.

Physical position based on sorghum genome v.3.

Total number of predicted genes within 55 kb of significant markers according to sorghum genome v.3.

Predicted N-terminal transit peptide targeting protein to mitochondria or chloroplast, according to TargetP-2.0 (Almagro Armenteros *et al.*, 2019).

Indicates if differentially expressed under pre-flowering drought stress in Tx642, Tx430, or both lines, according to Varoquaux *et al.* (2019).

## Discussion

Most sorghum studies dissecting the genetic control of drought tolerance have focused on post-flowering stages and the effect of the stay-green trait on grain yield. Stay-green lines delay senescence and sustain leaf function for a longer period, which generates higher yields under post-anthesis drought stress (Borrell *et al.*, 2000). Leaf senescence is a programmed process that can be hastened under environmental stress (Noodén *et al.*, 1997). In our study, drought was imposed pre-flowering (8–12 expanded leaves) to investigate a less characterized phenological stage, but the identification of 43 SNPs that are located within previously reported QTL for post-flowering stay-green supports the idea that common QTL could control drought responses across phenological stages, as previously demonstrated (Burke *et al.*, 2013) (Supplementary Table S8).

In quantitative genetic studies of drought stress response, it is particularly important to impose growing conditions that will ensure consistent growth rates and phenological states for the unbiased comparison of genotypes (Collins *et al.*, 2008). Similarly, the rate of progression and duration of the drought event must be homogenously applied across genotypes since they determine the type of stress response (Mcdonald and Davies, 1996). The automated irrigation system utilized in this study and the dry-down method imposed to reach the target drought level were effective to provide water stress to all plants, increasing the chances of identifying loci associated with drought tolerance, as suggested by Collins *et al.* (2008) and Blum (2011). Preliminary experiments conducted on a subset of lines (data not shown) demonstrated that 15% VWC was a target level that maximized the differential photosynthetic response of genotypes without inducing irreversible senescence that would have eliminated genotypes from the GWAS. Due to the broad genetic diversity of the SAP, it is possible that some genotypes (drought tolerant) experienced a moderate stress at the final VWC (15%), while others (more susceptible) suffered a severe stress at the same level. Therefore, the observed decrease in photosynthesis could be mostly due to stomatal limitations for some accessions but caused by photodamage in others.

The large range of variation in gas exchange and chlorophyll fluorescence variables observed in our study is in agreement with previous reports in sorghum (Kidambi *et al.*, 1990a; Peng *et al.*, 1991; Balota *et al.*, 2008; Guo *et al.*, 2008; Kiani *et al.*, 2008; Xin *et al.*, 2009; Salas Fernandez *et al.*, 2015; Sukumaran *et al.*, 2016; Ortiz *et al.*, 2017). The intermediate *h*^2^ of *A*, *E*, and *g*_s_ are similar to those described by other groups (Hervé *et al.*, 2001; Gu *et al.*, 2012; Ortiz *et al.*, 2017), and suggest that selection for genotypes with higher carbon fixation capacity is possible. The decline in *A* under drought stress was coupled with proportional reductions in *g*_s_ and *E*, which implies that genotypic differences in photosynthetic performance are mostly associated with stomatal closure, as noted by Cornic (2017). Genotypic effects for *A*:*E* were not significant during the drought and recovery treatments, with narrower phenotypic variation than previously reported (Fig. 2) (Balota *et al.*, 2008). The non-stress *F*_v_/*F*_m_ values are as expected for a C_4_ species (Björkman and Demmig, 1987; Fracheboud *et al.*, 1999; Cousins *et al.*, 2002) and the decline under drought and recovery treatments is indicative of the typical photoinhibition of stressed plants. The reduction in Φ_PSII_ during drought and recovery can be explained by decreases in both energy capture (*F*_v_ʹ/*F*_m_ʹ) and the fraction of open reaction centers (qP). The variability in the ratio of control to drought for *F*_v_ʹ/*F*_m_ʹ suggests that genotypes had large differences in non-photochemical quenching (NPQ) capacity, as expected (Zegada-Lizarazu and Monti, 2013), which could be exploited for the development of drought-tolerant germplasm.

### Regions co-localizing with previously reported drought response or photosynthesis-related QTL

The large number of significant SNPs/genomic regions explaining small percetanges of the phenotypic variation (*R*^2^) are consistent with the complex genetic architecture of photosynthetic traits (Table 1), which has been described in other gas exchange and chlorophyll fluorescence studies under non-stress and abiotic stress (Fracheboud *et al.*, 2002; Hao *et al.*, 2012; Strigens *et al.*, 2013; van Rooijen *et al.*, 2015), including sorghum in response to cold (Fiedler *et al.*, 2014; Ortiz *et al.*, 2017). Therefore, we prioritized discoveries based on the coincident localization with previously reported QTL for agronomic performance under drought or photosynthesis-related traits under abiotic stress, which provides an initial independent validation that could be subsequently reinforced with functional studies. Eight regions on chromosomes 1, 4, and 7 co-localized with QTL related to chlorophyll fluorescence, *E*, Φ_PSII_, *g*_*s*_, *A*, chlorophyll content, dry matter growth rate, green leaf area, number of green leaves, water-use efficiency, and grain yield under drought stress. All these regions were discovered due to variation observed during the recovery period, except one (region 4_7) that was correlated with parameters quantified during drought or across periods (Table 2; Fig. 3).

The number of predicted genes in each of these eight prioritized regions (Fig. 4) varied from six to 56, but only 22 were differentially expressed in either Tx642, Tx430, or both lines during pre-flowering drought stress according to Varoquaux *et al.* (2019). The presence of a predicted N-terminal peptide pre-sequence for the subcellular localization to mitochondria or chloroplasts was detected in 10 of these 22 most promising candidates for future studies and genetic manipulation (Table 2).

In region 1_9, two promising candidates are predicted to encode proteins targeted to the chloroplast (Table 2). *Sobic.001G248300* is homologous to acylamino acid-releasing enzymes (AAREs) (58% identity to *At4g14570*), which are involved in the turnover and destabilization of acetylated proteins, such as Rubisco, under oxidative conditions in Arabidopsis (Yamauchi *et al.*, 2003). Glutathione reductases are involved in scavenging reactive oxygen species (ROS) through the ascorbate–glutathione cycle and are essential to maintain efficient photosynthesis particularly under stress (Müller-Schüssele *et al.*, 2020). *Sobic.001G248500* is homologous to Arabidopsis *GR2* (*At3g54660*, 66% identity), which is essential in the plastid stroma (Marty *et al.*, 2019), protecting PSII from excess light and maintaining the repair of photodamaged PSII (Ding *et al.*, 2016).

One gene is singled out from regions 1_8, 1_20, and 7_3, considering the previously reported differential expression under drought, but none of them contains chloroplast or mitochondria targeting signals (Table 2). Endoplasmic reticulum-type Ca^2+^-transporting ATPases (predicted function for *Sobic.001G103300*) are required and up-regulated to maintain low Ca^2+^ cytosolic levels by sequestering ions to intracellular compartments, which are used by plants to trigger signaling pathways in response to abiotic stress (Knight *et al.*, 1997; Aslam *et al.*, 2017). *Sobic.001G286100* is similar (84% identity) to an inositol 1,3,4-trisphosphate 5/6 kinase (ITPK). The overexpression of a rice ITPK homolog generated a drought- and salt-hypersensitive response, with a decrease in inositol trisphosphate and down-regulation of ROS-scavenging genes (Du *et al.*, 2011). Under abiotic stress, chlorophyll molecules are released from thylakoid membranes and should be degraded quickly to avoid damage induced by their photodynamic capacity (Takamiya *et al.*, 2000). Chlorophyllases catalyze this breakdown of chlorophyll but not as a consequence of senescence. *Sobic.007G168000* is predicted as a chlorophyllase-1, homolog of *At1g19670*, which has been associated with the degradation of chlorophyll, and whose silencing caused accumulation of hydrogen peroxide under high light conditions and induction of antioxidant mechanisms (Kariola *et al.*, 2005).

Three promising candidates were identified in region 4_7 (Table 2). *Sobic.004G281600* is predicted to encode a mitochondrial mechanosensitive ion channel protein with 54% identity to Arabidopsis *MSL1* (*At4g00290*). If the mitochondria membrane potential is too high, the ROS production and lipid peroxidation are increased. Ion transport mediated by *MSL1* reduced the mitochondrial membrane potential under stress, as demonstrated by the increased oxidation of the mitochondria glutathione pool in *MSL1* knockout mutants (Lee *et al.*, 2016). *Sobic.004G282000* contains a pentatricopeptide repeat (PPR) and the highest homology to several members of PPR-containing proteins in Arabidopsis that act in RNA editing which occurs in both chloroplasts and mitochondria (*At2g13600* and *At1g11290*). As an example, a mutation in *SLO2* (*At2g13600*) caused hypersensitivity to abscisic acid (ABA), accumulation of ROS, and increased drought tolerance (Zhu *et al.*, 2014), while inducing changes in RNA editing at seven sites of proteins belonging to complex I of the mitochondrial electron transport chain (Zhu *et al.*, 2012). Chloroplast PPR genes have also been associated with RNA editing and photosynthetic capacity, as demonstrated by the knockout mutation of *OsPPR16*, which edited *rpoB* RNA of the plastid-encoded RNA polymerase, affecting chlorophyll synthesis and normal chloroplast development (Huang *et al.*, 2020). *Sobic.004G282400* encodes a Rho GTPase-activating protein homologous to the product of *At3g11490* (62% identity), whose knockout mutants expressed an increased sensitivity to stress induced by oxygen deprivation.

Another region on chromosome 4 (4_2) harbors a homolog of *At3g20290* (*Sobic.004G047100*) which is predicted to encode an AtEHD1 protein required for endocytosis and membrane trafficking of recycling endosomes (Bar *et al.*, 2013). Altering the balance between exo- and endocytic protein trafficking, particularly of K^+^ ion channels, affects adaptation to environmental clues (Bar *et al.*, 2013; Zhao *et al.*, 2017), as demonstrated by the defective stomatal movement caused by a reduction in endocytosis leading to dehydration tolerance (Larson *et al.*, 2017; Zhang *et al.*, 2019). Two cyclases in this region (*Sobic.004G069850* and *Sobic.004G070000*) had 64–79% identity to Arabidopsis cyclase genes *CYL1*, *CYL2*, and *CYL3*, a family that has been associated with the accumulation of ROS and response to multiple abiotic stresses (Qin *et al.*, 2015). *Sobic.004G070200* encodes a protein targeted to the chloroplast that is involved in rRNA and mRNA maturation (65% identity to *At5g63420*). Silencing of *At5g63420* caused chlorosis and revealed its role in the normal formation of thylakoid membranes (Chen *et al.*, 2015) and the reduction of RNA antisense–sense duplexes affecting chloroplast RNA quality (Hotto *et al.*, 2020) (Table 2).

An RNA helicase (*Sobic.001G126500*) was singled out in region 1_15, due to the known role of helicases in ribosome biogenesis and abiotic stress tolerance (Liu and Imai, 2018), particularly affecting photosynthesis and the antioxidant machinery (Tuteja *et al.*, 2013; Nawaz and Kang, 2019). A predicted chloroplast-targeted protein (*Sobic.001G128600*) with an unknown function is a homolog to the product of *At1g54520* (62% identity), which was identified as part of the chloroplast envelope and stroma–-lamella fraction, differentially phosphorylated in response to *STN8*. The transition from cyclic to linear electron flow upon illumination is mediated by *STN8* (Reiland *et al.*, 2011). *Sobic.001G129700* encodes a germin-like (oxalate oxidase) protein (77% identity to P15290 from wheat) that was differentially expressed in an osmotic stress study using sorghum cell cultures (Ashwin Ngara *et al.*, 2018). Tobacco plants overexpressing oxalate oxidase from wheat had a higher H_2_O_2_ concentration, higher expression of antioxidant enzyme genes, and increased tolerance to oxidative stress induced by high light (Hunt and Gray, 2009). Epidermal patterning factors (EPFs) are regulators of stomatal development, affecting density and spacing. *Sobic.001G140400* is a homolog to EPF-like2 (48% identity to NP_680774), and overexpression of EPFL genes leads to reduced stomatal density and increased drought tolerance in rice and Arabidopsis (Wan *et al.*, 2009; Caine *et al.*, 2019). Finally, pyruvate kinases, which catalyze the last step of glycolysis, are differentially regulated by drought in many tissues. *Sobic.001G140700* is a chloroplast-targeted pyruvate kinase, which could be responding to changes in oxygen levels due to stomatal closing, and compensanting for energy losses, as previously reported in barley (Janiak *et al.*, 2018) (Table 2).

### New loci associated with photosynthetic response during the recovery period

Five LD regions on chromosomes 1, 2, and 5 were associated with gas exchange and/or chlorophyll fluorescence parameters and did not localize with previously reported QTL for agronomic performance under drought or photosynthesis-related traits (Table 3). All regions explained variation during the recovery period and harbored a total of 64 predicted genes. Twenty of these candidates were differentially expressed under drought (Varoquaux *et al.*, 2019) and represent the most promising targets for future functional validation and manipulation to improve drought tolerance.

On chromosome 1 (regions 1_22 and 1_23), *Sobic.001G300201* encodes a basic helix–loop–helix (bHLH) transcription factor with homology to *ZmPIF1* (GRMZM2G115960) (63% identity). Transgenic rice and Arabidopsis expressing *ZmPIF1* exhibited drought resistance due to a reduced stomatal conductance and transpiration mediated by ABA (Gao *et al.*, 2018). Expansins (EXPs), a predicted function of *Sobic.001G300700*, are cell wall proteins involved in cell growth and reported to induce drought tolerance in wheat (Zhao *et al.*, 2011). Overexpression of wheat *EXPB23* led to transgenic tobacco plants that lost water more slowly, and maintain better cell structure and superior photosynthetic performance under drought stress (Li *et al.*, 2011). Similar results were obtained in sugarcane and potato (Chen *et al.*, 2019; Ashwin Narayan *et al.*, 2021). *Sobic.001G302300* encodes a protein of unknown function but with low homology to SNOWY COTYLEDON 3 (SCO3) protein in Arabidopsis (*At3g19570*; 28% identity). This gene that belongs to a large uncharacterized family unique to plants is required for normal chloroplast biogenesis and its mutation reduces chlorophyll accumulation, thylakoid formation, and photosynthesis (Albrecht *et al.*, 2010) (Table 3).

In region 2_3, *Sobic.002G199900* is predicted to encode a protein with homology to the plant peptide-containing tyrosine sulfation (PSY) family (Table 3). These small peptides are post-translationally modified and partially regulated at the transcriptional level, associated with the modulation of ABA responses to abiotic stress (Tost *et al.*, 2021). *Sobic.002G200200*, predicted to encode a chloroplast-targeted protein with homology to CaaX-like (59% identity to *At1g14270*), is a promising candidate because a CaaX-like endopeptidase (*SCO4* gene) was involved in the acclimation of chloroplasts and their photosystems to excesss light in Arabidopsis. *SCO4* mutants showed a decreased in linear electron transfer, reduced function of PSI, and increased NPQ (Albrecht-Borth *et al.*, 2013).

Out of the 13 genes localized in region 5_4, two were differentially expressed under drought conditions, but only *Sobic.005G202600* has a predicted function related to our traits (Table 3). It has homology to a senescence-associated protein 13 (SAG13) (74% identity to *At2g29350*), which is a small alcohol dehydrogenase/oxidoreductase that responds quickly and strongly to ABA treatments, not only in older leaves or as an indirect response to senescence (Weaver *et al.*, 1998).

The last newly associated region 5_5 harbored the largest number of genes, with 11 of them reported as differentially expressed in response to drought (Varoquaux *et al.*, 2019). *Sobic.005G229000* is one of the most promising candidates based on its homology to SC35-like splicing factor 30 (79% identity to *At3g55460*), which is an RNA-binding protein, part of the spliceosome that is responsive to drought stress (Marondedze *et al.*, 2020). Interestingly, a tandem of six GRAS-type transcription factors were localized in this region (Table 3). In Arabidopsis, this transcription factor family has been associated with numerous growth and development functions, having redundant roles (Lee *et al.*, 2008). In rice, *OsGRAS23* was located in a drought-resistant QTL, and, when overexpressed, plants showed drought resistance and tolerance to oxidative stress. Several antioxidation genes were also up-regulated in these transgenic plants (Xu *et al.*, 2015).

## Concluding remarks

Our study aimed to characterize and exploit the sorghum natural variation in photosynthetic performance under optimal growing conditions, in response to drought and during the subsequent recovery to elucidate the genetic architecture of these important traits, and ultimately identify candidate genes for genetic improvement, engineering, or editing. The response to abiotic stress is complex in nature and involves the expression of multiple genes with small effects. To address this challenge, the GWAS presented herein was conducted using a large number of accessions and was complemented by comparative studies with previously reported QTL of related traits, public RNAseq data, and prediction of organelle targeting peptide signals. The most important discoveries are: (i) there is large natural variation in gas exchange and chlorophyll fluorescence traits in response to non-stress, drought, and recovery conditions; (ii) stomatal control was the main factor explaining reductions in gas exchange under drought and recovery treatments; (iii) sorghum lines presented a large *A*:*E* range under optimal growing conditions but minimal variation under drought and recovery; (iv) the majority of SNP–trait associations corresponded to the recovery treatment, revealing the importance of exploiting post-stress adaptation mechanisms; (v) 39 significant SNPs are co-localized with previously reported QTL for stay-green in sorghum even though drought stress was imposed during pre-flowering; and (vi) 22 promising candidate genes were identified based on multiple layers of selection and homology to genes with functions related to photosynthesis and photoprotection. Finally, our results represent a valuable resource to select genotypes with enhanced tolerance to drought stress. The exploration of syntenic regions with other species such as maize, wheat, and rice could also support breeding efforts to enhance carbon fixation capacity in other commercially important crops.

## Supplementary data

The following supplementary data are available at *JXB* online.

Fig. S1. Photosynthesis (*A*) as a function of stomatal conductance (*g*_s_) in sorghum in control (30% VWC), drought (15 % VWC), and recovery periods (30% VWC).

Fig. S2. Photosynthesis (*A*) as a function of effective quantum yield of PSII (ΦPSII) in sorghum in control (30% VWC), drought (15 % VWC), and recovery periods (30% VWC).

Fig. S3. Genome-wide association study results for *A* in three soil water content treatments and in cumulative response, drought/control ratio, and drought/recovery ratio using 324 diverse sorghum accessions.

Fig. S4. Genome-wide association study results for *E* in three soil water content treatments and in cumulative response, drought/control ratio, and drought/recovery ratio using 324 diverse sorghum accessions.

Fig. S5. Genome-wide association study results for *g*_s_ in three soil water content treatments and in cumulative response, drought/control ratio, and drought/recovery ratio using 324 diverse sorghum accessions.

Fig. S6. Genome-wide association study results for *F*_v_/*F*_m_ in three soil water content treatments and in cumulative response, drought/control ratio, and drought/recovery ratio using 324 diverse sorghum accessions.

Fig. S7. Genome-wide association study results for *F*_v_ʹ/*F*_m_ʹ in three soil water content treatments and in cumulative response, drought/control ratio, and drought/recovery ratio using 324 diverse sorghum accessions.

Fig. S8. Genome-wide association study results for Φ_PSII_ in three soil water content treatments and in cumulative response, drough/control ratio, and drought/recovery ratio using 324 diverse sorghum accessions.

Fig. S9. Genome-wide association study results for qP in three soil water content treatments and in cumulative response, drought/control ratio, and drought/recovery using 324 diverse sorghum accessions.

Fig. S10. Genome-wide association study results for *A*:*E* ratio in three soil water content treatments using 324 diverse sorghum accessions.

Table S1. Soil water content (VWC) of 324 accessions averaged across the last 3 d of drought treatment.

Table S2. Phenotypic correlations between photosynthesis and chlorophyll fluorescence traits based on BLUPs in the control period.

Table S3. Phenotypic correlations between photosynthetic and chlorophyll fluorescence traits based on BLUPs in the drought period.

Table S4. Phenotypic correlations between photosynthetic and chlorophyll fluorescence traits based on BLUPs in the recovery period.

Table S5. ANOVA of photosynthesis and chlorophyll fluorescence traits in control, drought, and recovery periods.

Table S6. Summary of GWAs results for gas exchange and chlorophyll fluorescence traits in control, drought, and recovery periods, derived variables cumulative response, and drought/recovery ratio.

Table S7. Complete list of sorghum genes localized ±55 kb of significant SNPs.

Table S8. Summary of associated regions co-localizing with previously published QTL for drought stress response and/or photosynthesis-related traits.

Table S9. Subset of associated LD regions for both gas exchange and chlorophyll fluorescence traits co-localized with QTL for drought response or photosynthesis-related traits.

## Supplementary Material

erab502_suppl_Supplementary_Figures_S1-S10_Tables_S1-S6

erab502_suppl_Supplementary_Table_S7

erab502_suppl_Supplementary_Table_S8

erab502_suppl_Supplementary_Table_S9

## Data Availability

Genotypic data utilized for GWAS are publicly available at https://www.morrislab.org/data. The phenotypic data supporting findings of this study are available from the corresponding author, Maria G. Salas-Fernandez, upon request.

## Abbreviations:

*A*: leaf carbon assimilation rate
*E*: transpiration rate
*F*_v_/*F*_m_: maximum quantum yield of PSII
*F*_v_ʹ/*F*_m_ʹ: efficiency of energy captured by open PSII reaction centers
*g*_s_: stomatal conductance
GWAS: genome-wide association study
LD: linkage disequilibrium
LHC: light-harvesting complex
qP: photochemical quenching or fraction of PSII reaction centers that are open
QTL: quantitative trait locus
SNP: single nucleotide polymorphism
Φ_PSII_: effective quantum yield of PSII
