# Supplementary material for: Dissecting the genetic control of natural variation in sorghum photosynthetic response to drought stress
Source: J Exp Bot. 2021 Nov 16;73(10):3251–67. doi: 10.1093/jxb/erab502 (PMC9126735; doi:10.1093/jxb/erab502)
Supplement: erab502_suppl_Supplementary_Figures_S1-S10_Tables_S1-S6 [file erab502_suppl_supplementary_figures_s1-s10_tables_s1-s6.pdf]

Dissecting the genetic control of natural variation in sorghum photosynthetic response to drought stress

Diego Ortiz, Maria G. Salas-Fernandez

Supplementary figures

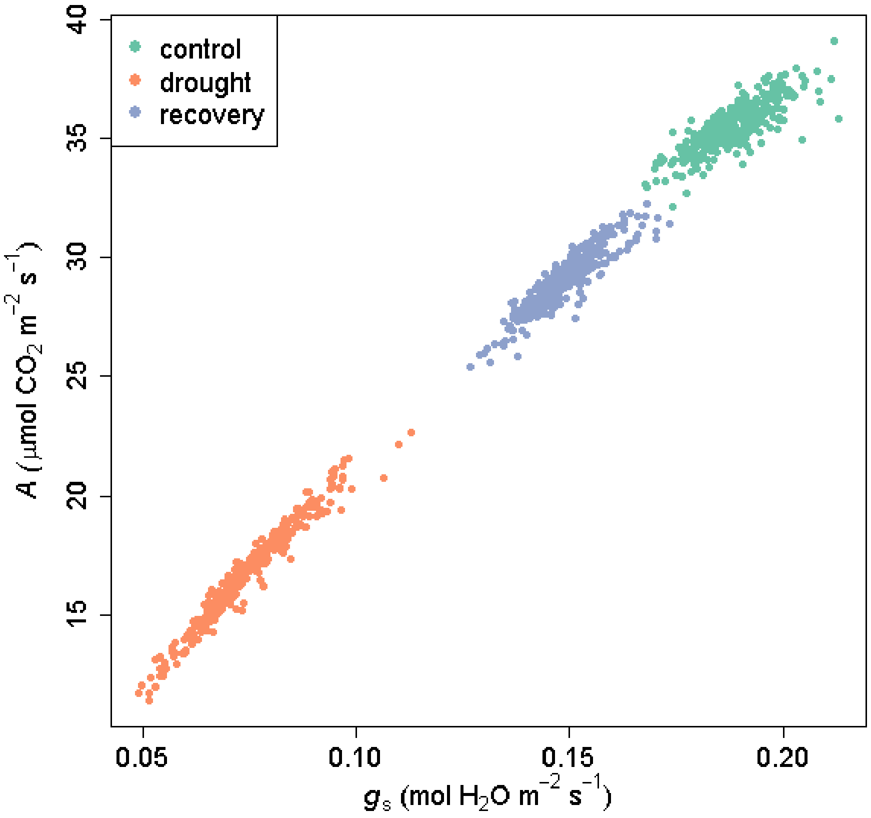

Supplementary Figure S1. Photosynthesis (A) as a function of stomatal conductance (g_s) in sorghum in control (30% VWC), drought (15 % VWC), and recovery periods (30% VWC). All measurements were performed on fully expanded leaves from 30-day old plants grown in 6-L pots.

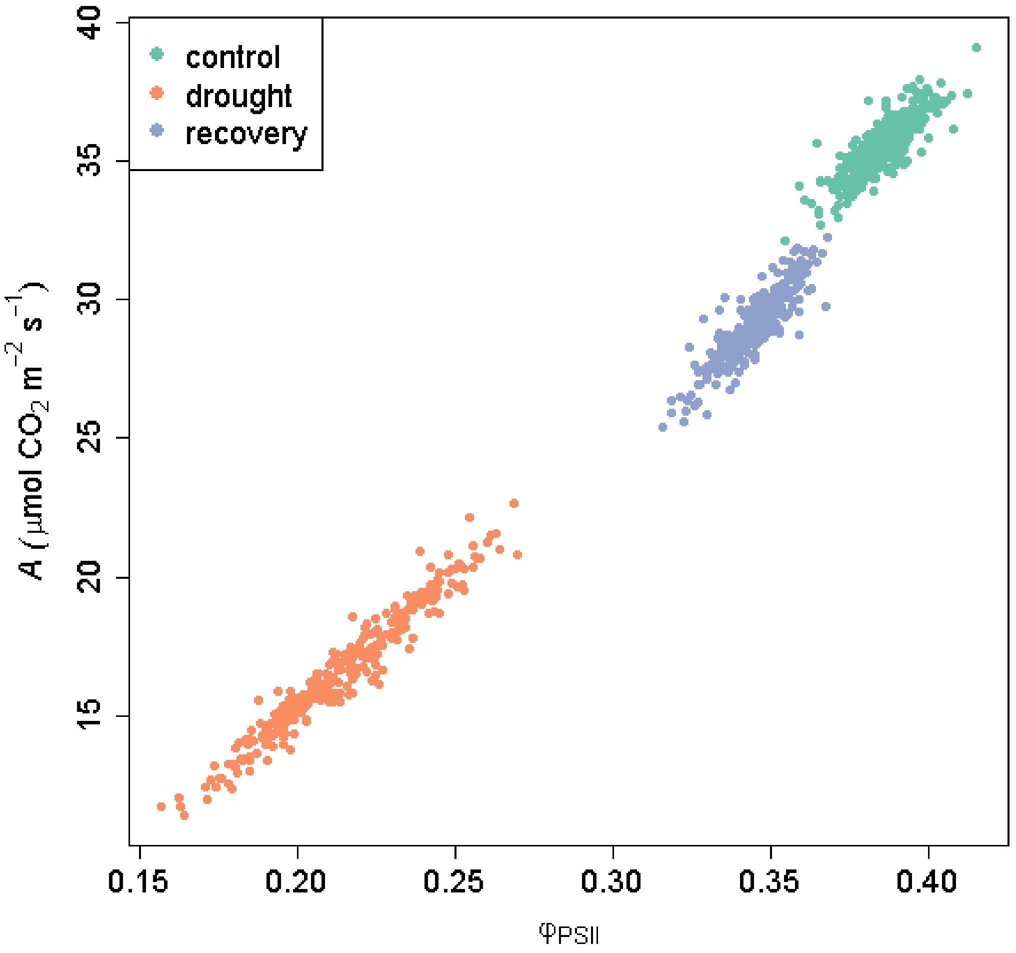

Supplementary Figure S2. Photosynthesis (*A*) as a function of effective quantum yield of PSII (ΦPSII) in sorghum in control (30% VWC), drought (15 % VWC), and recovery periods (30% VWC). All measurements were performed on fully expanded leaves from 30-day old plants grown in 6-L pots.

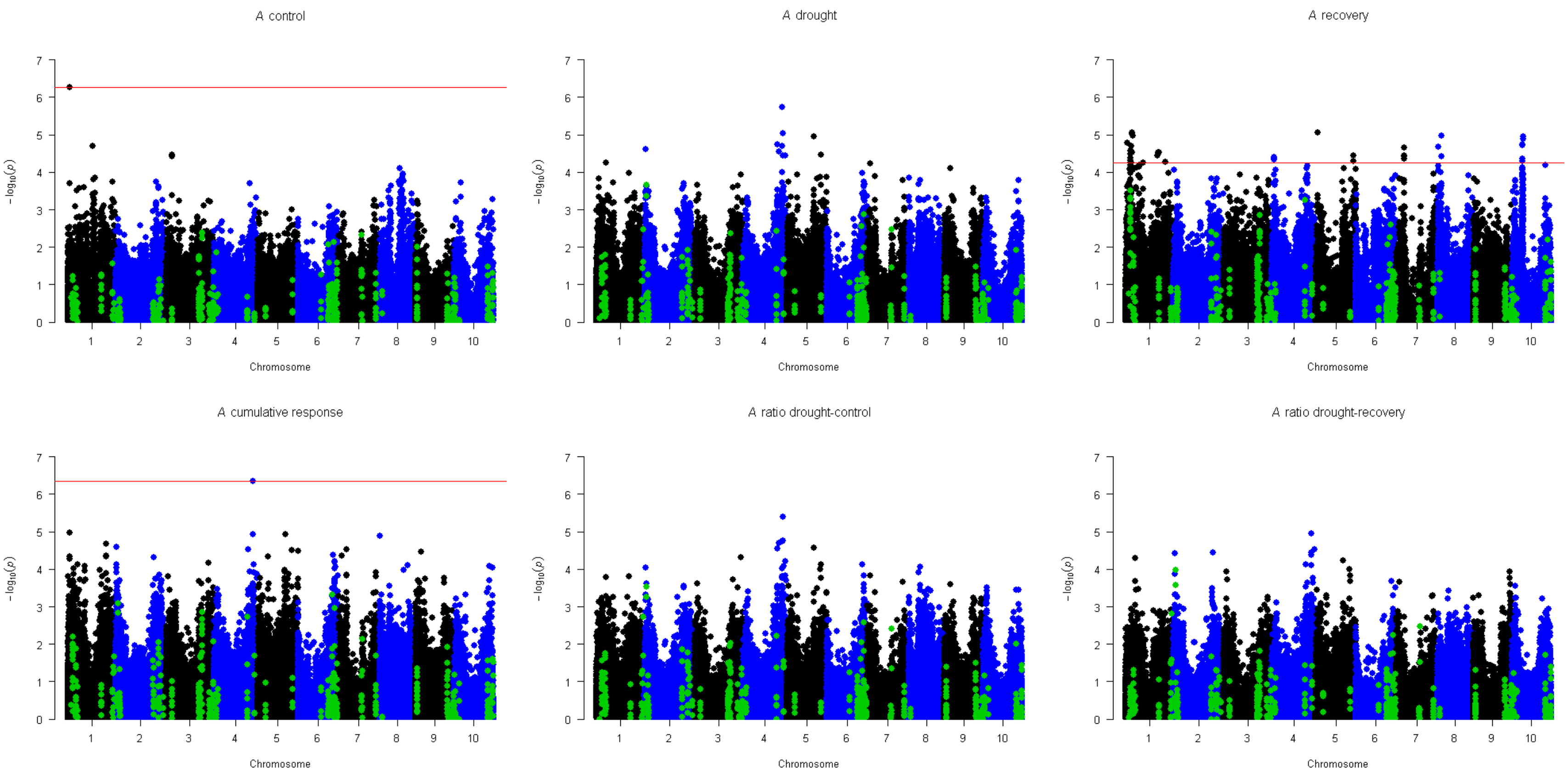

Supplementary Figure S3. Genome-wide association study results for *A* in three soil water content treatments and in cumulative response, ratio drought-control and ratio drought-recovery using 324 diverse sorghum accessions. Horizontal red line indicates significance threshold. Green dots indicate the physical position of *a priori* candidate genes. Each single-nucleotide polymorphism is represented by a dot, whose center indicates the exact physical position of the marker.

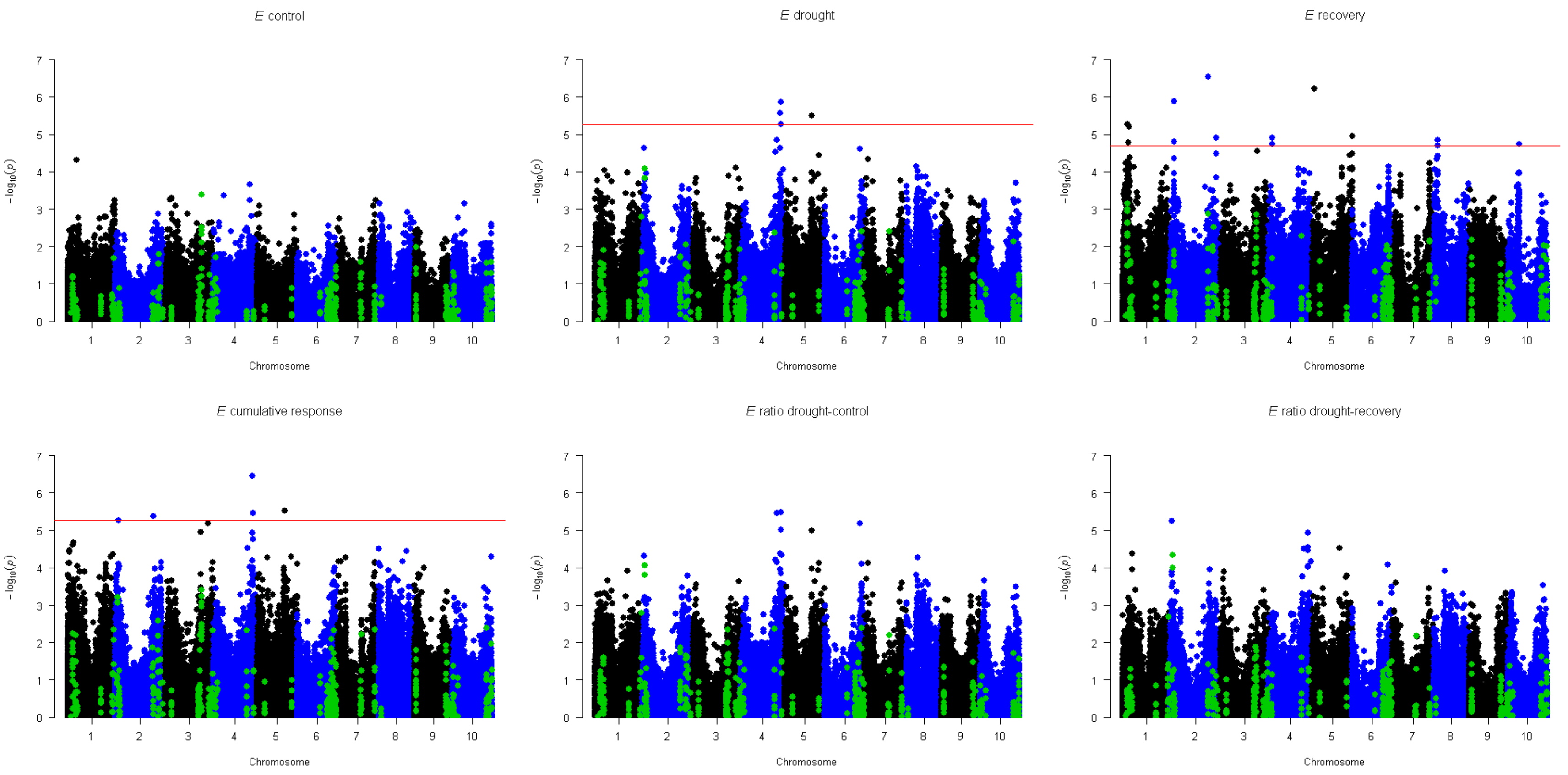

Supplementary Figure S4. Genome-wide association study results for *E* in three soil water content treatments and in cumulative response, ratio drought-control and ratio drought-recovery using 324 diverse sorghum accessions. Horizontal red line indicates significance threshold. Green dots indicate the physical position of *a priori* candidate genes. Each single-nucleotide polymorphism is represented by a dot, whose center indicates the exact physical position of the marker.

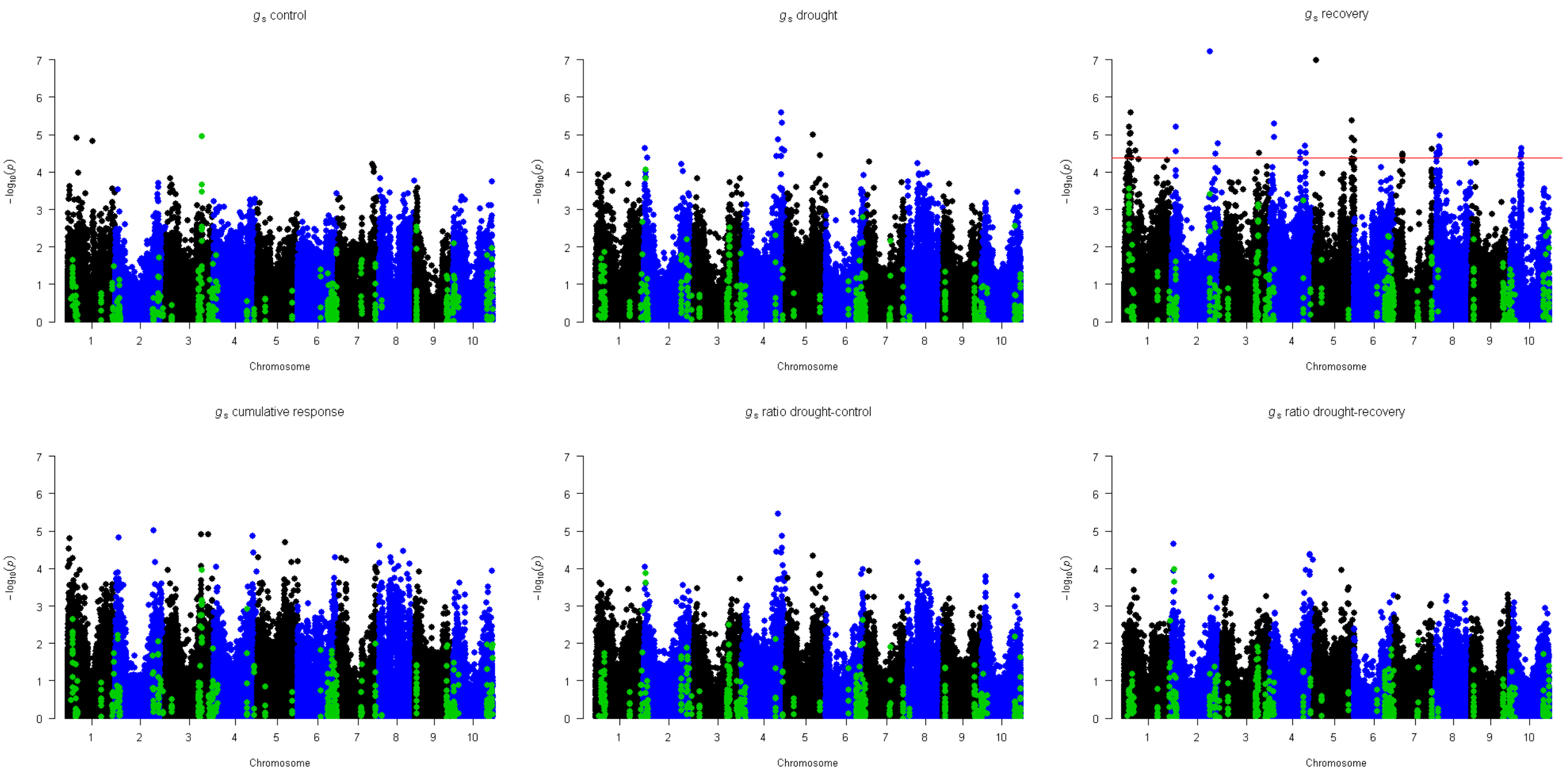

Supplementary Figure S5. Genome-wide association study results for g_s in three soil water content treatments and in cumulative response, ratio drought-control and ratio drought-recovery using 324 diverse sorghum accessions. Horizontal red line indicates significance threshold. Green dots indicate the physical position of *a priori* candidate genes. Each single-nucleotide polymorphism is represented by a dot, whose center indicates the exact physical position of the marker.

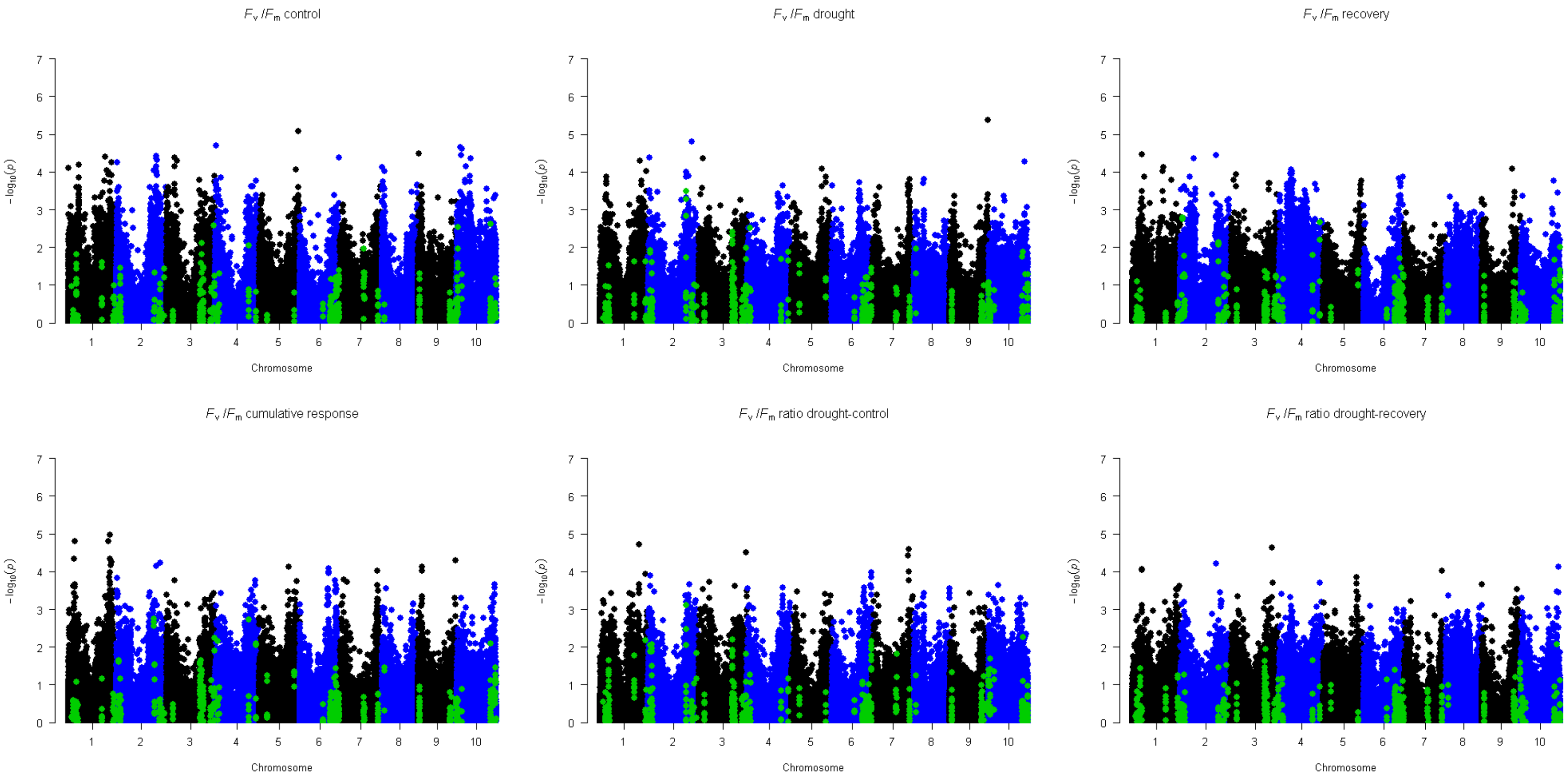

Supplementary Figure S6. Genome-wide association study results for F_v/F_m in three soil water content treatments and in cumulative response, ratio drought-control and ratio drought-recovery using 324 diverse sorghum accessions. Horizontal red line indicates significance threshold. Green dots indicate the physical position of *a priori* candidate genes. Each single-nucleotide polymorphism is represented by a dot, whose center indicates the exact physical position of the marker.

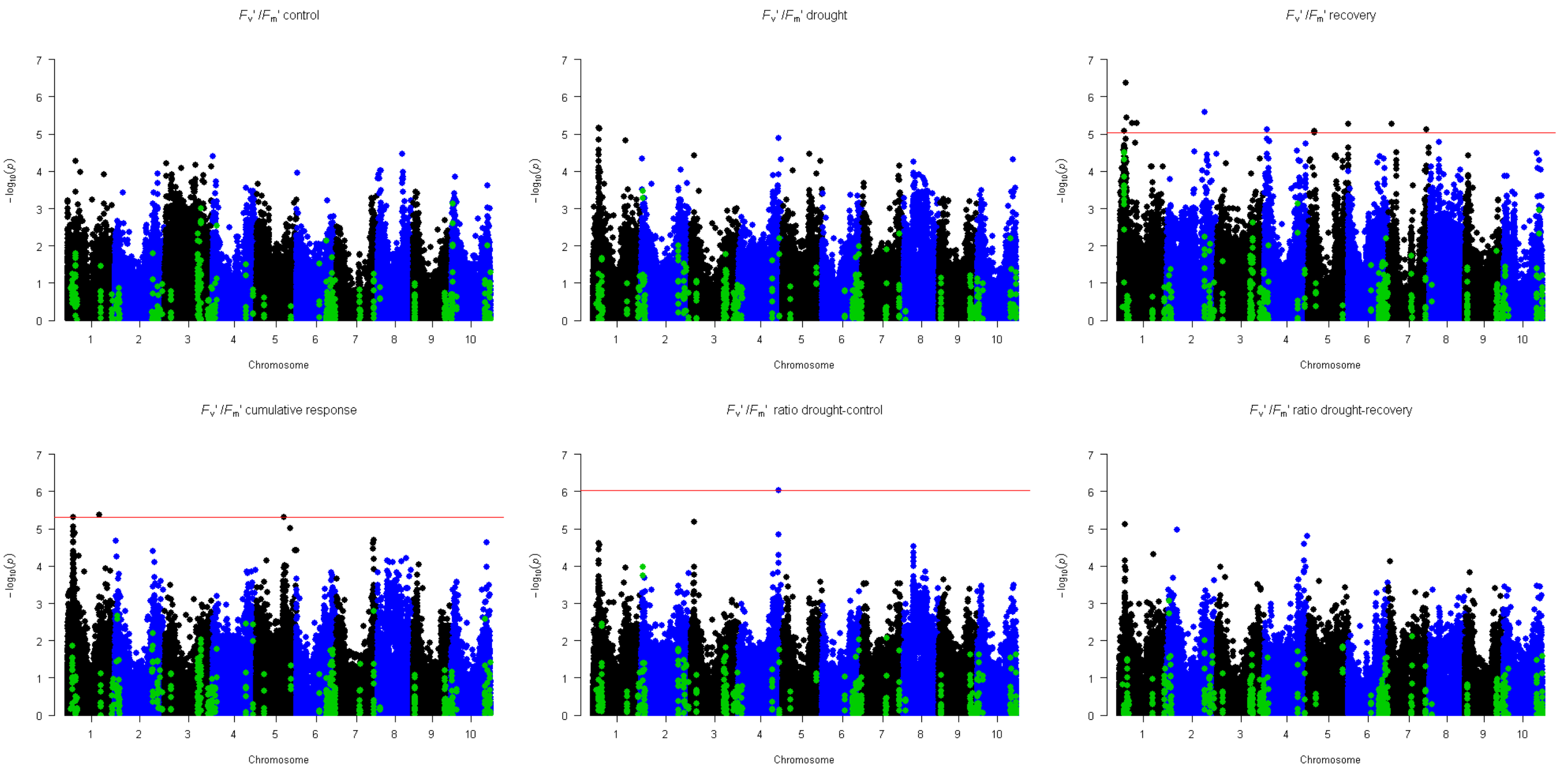

Supplementary Figure S7. Genome-wide association study results for F_v'/F_m' in three soil water content treatments and in cumulative response, ratio drought-control and ratio drought-recovery using 324 diverse sorghum accessions. Horizontal red line indicates significance threshold. Green dots indicate the physical position of *a priori* candidate genes. Each single-nucleotide polymorphism is represented by a dot, whose center indicates the exact physical position of the marker.

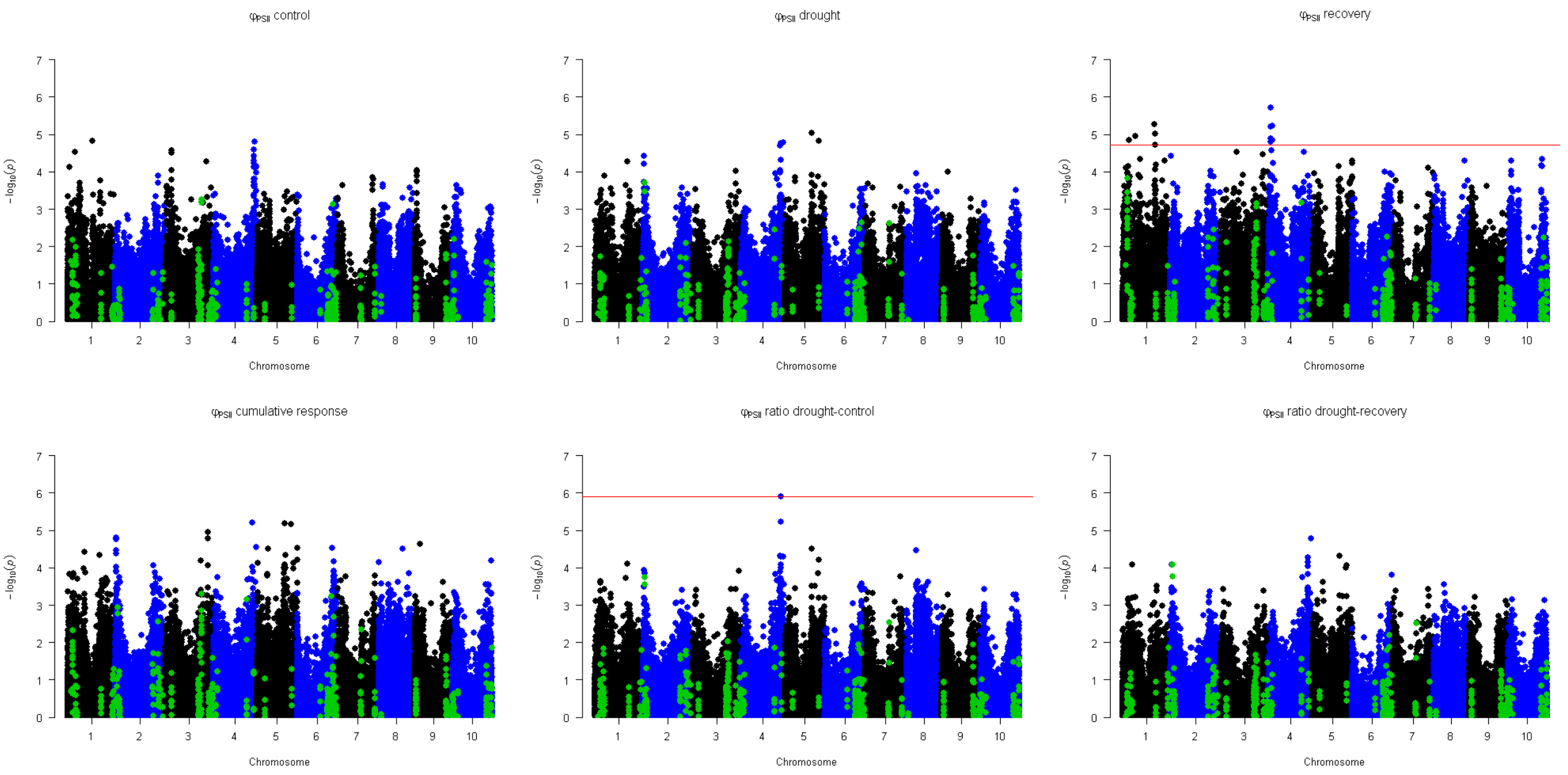

Supplementary Figure S8. Genome-wide association study results for φ_{PSII} in three soil water content treatments and in cumulative response, ratio drought-control and ratio drought-recovery using 324 diverse sorghum accessions. Horizontal red line indicates significance threshold. Green dots indicate the physical position of *a priori* candidate genes. Each single-nucleotide polymorphism is represented by a dot, whose center indicates the exact physical position of the marker.

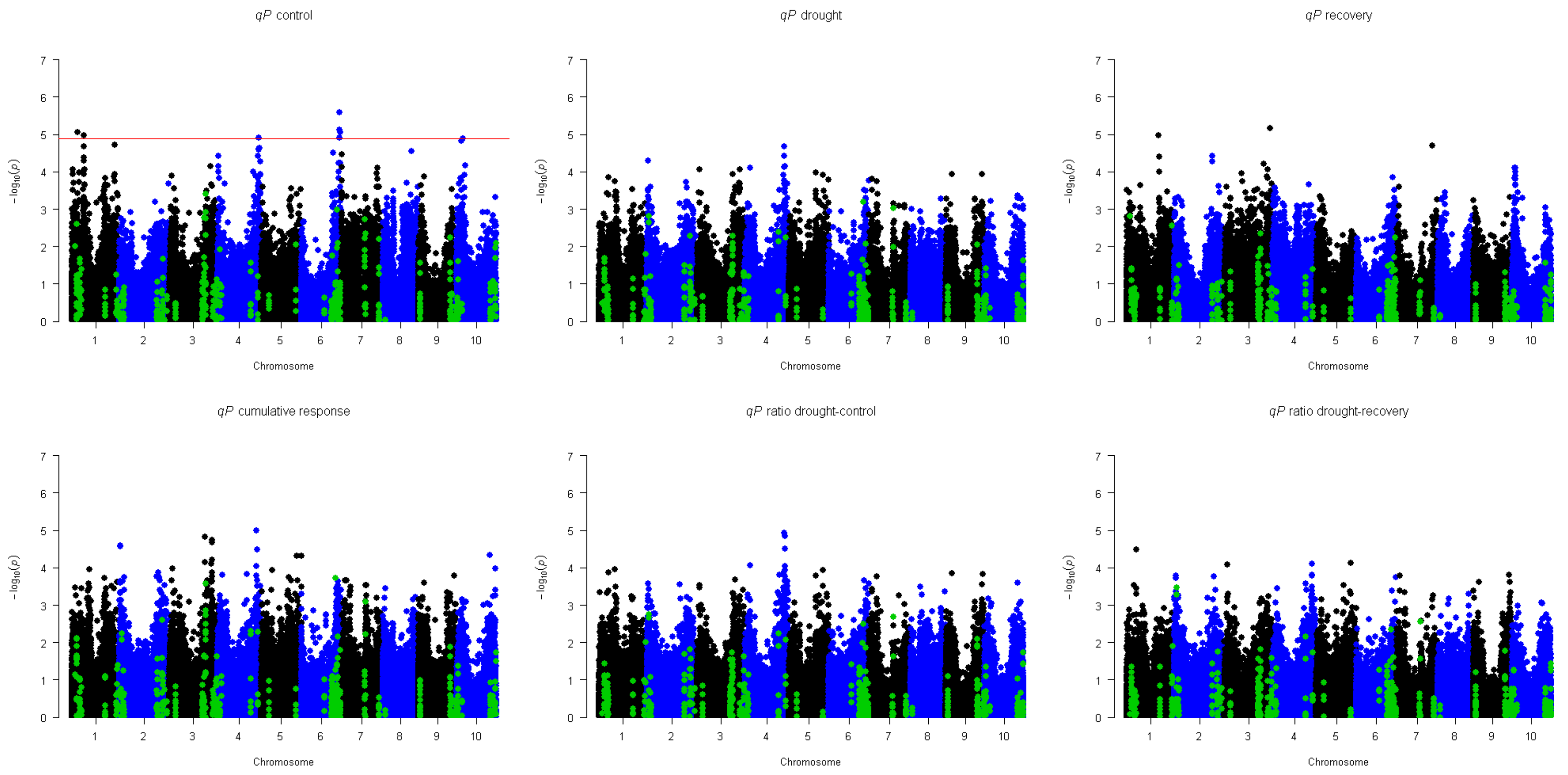

Supplementary Figure S9. Genome-wide association study results for *qP* in three soil water content treatments and in cumulative response, ratio drought-control and ratio drought-recovery using 324 diverse sorghum accessions. Horizontal red line indicates significance threshold. Green dots indicate the physical position of *a priori* candidate genes. Each single-nucleotide polymorphism is represented by a dot, whose center indicates the exact physical position of the marker.

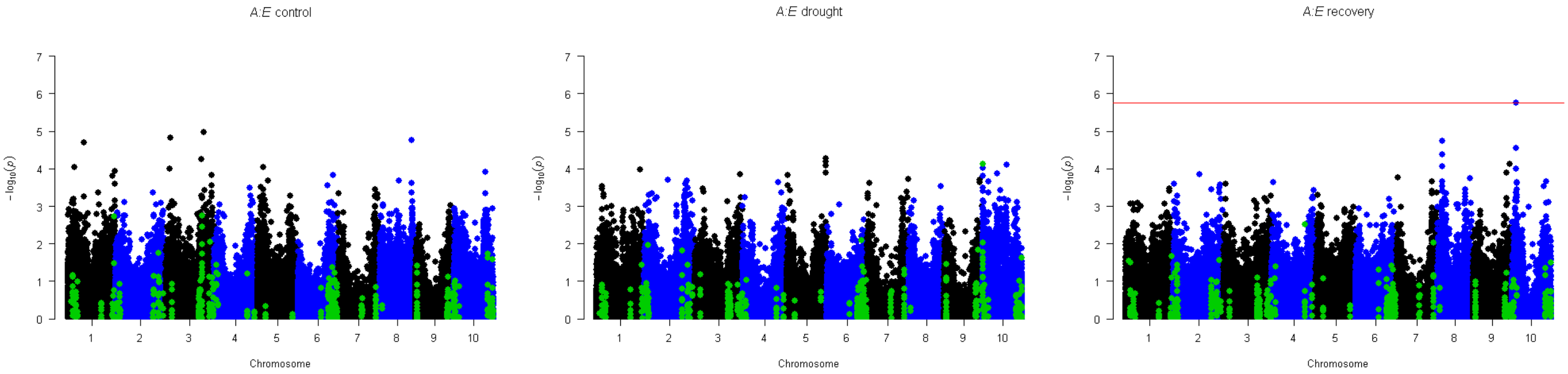

Supplementary Figure S10. Genome-wide association study results for *A:E* ratio in three soil water content treatments using 324 diverse sorghum accessions. Horizontal red line indicates significance threshold. Green dots indicate the physical position of *a priori* candidate genes. Each single-nucleotide polymorphism is represented by a dot, whose center indicates the exact physical position of the marker.

Supplemental Table S1. Soil water content (VWC) of 324 accessions averaged across the last 3 days of drought treatment

	Average VWC		Standard Deviation		Coefficient of variation	
Accession	Rep 1	Rep 2	Rep 1	Rep 2	Rep 1	Rep 2
PI656029	0.176	0.157	0.023	0.046	13.010	29.408
PI656029	0.186	0.178	0.030	0.032	16.170	17.850
PI656029	0.171	0.176	0.018	0.018	10.217	9.991
PI656029	0.175	0.172	0.022	0.021	12.830	12.356
PI656029	0.183	0.150	0.028	0.023	15.390	15.313
PI656029	0.174	0.202	0.022	0.031	12.738	15.442
PI656029	0.175	0.167	0.019	0.016	10.812	9.806
PI656029	0.201	0.181	0.034	0.025	16.771	13.595
PI656029	0.186	0.189	0.028	0.024	15.225	12.786
PI655996	0.160	0.184	0.014	0.039	8.716	21.397
PI655996	0.177	0.178	0.019	0.025	10.740	14.151
PI655996	0.173	0.164	0.017	0.023	9.873	14.173
PI655996	0.186	0.175	0.030	0.034	16.014	19.216
PI655996	0.199	0.201	0.031	0.031	15.611	15.292
PI655996	0.179	0.176	0.026	0.016	14.706	9.206
PI655996	0.176	0.173	0.021	0.022	12.173	12.578
PI655996	0.194	0.185	0.028	0.021	14.554	11.241
PI655996	0.180	0.183	0.020	0.021	11.068	11.692
PI533839	0.161	0.173	0.019	0.038	11.938	21.740
PI533839	0.181	0.168	0.025	0.025	13.963	14.658
PI533839	0.180	0.165	0.023	0.018	12.744	10.767
PI533839	0.182	0.178	0.029	0.027	15.927	14.920
PI533839	0.182	0.196	0.042	0.026	23.019	13.273
PI533839	0.190	0.208	0.011	0.024	5.851	11.426
PI533839	0.166	0.179	0.016	0.024	9.695	13.437
PI533839	0.175	0.171	0.027	0.040	15.362	23.484
PI533839	0.187	0.186	0.026	0.021	13.895	11.467
PI564163	0.161	0.177	0.019	0.046	11.534	25.836
PI564163	0.181	0.160	0.028	0.018	15.601	11.276
PI564163	0.184	0.184	0.025	0.028	13.371	15.453
PI564163	0.183	0.165	0.028	0.033	15.142	19.837
PI564163	0.172	0.168	0.033	0.030	19.019	17.677
PI564163	0.167	0.188	0.014	0.027	8.180	14.105
PI564163	0.173	0.181	0.019	0.022	11.132	12.036
PI564163	0.178	0.192	0.026	0.029	14.702	15.392
PI564163	0.192	0.189	0.030	0.031	15.390	16.354
PI533821	0.159	0.150	0.024	0.034	14.934	22.635
PI655977	0.193	0.174	0.035	0.037	17.948	21.453
PI597952	0.183	0.157	0.030	0.026	16.175	16.239
PI534105	0.166	0.193	0.027	0.025	16.266	13.067
PI533913	0.166	0.189	0.014	0.038	8.618	20.020
PI656110	0.158	0.179	0.034	0.039	21.747	21.957

PI533919	0.159	0.179	0.012	0.038	7.794	21.394
PI656113	0.157	0.165	0.016	0.020	10.252	12.134
PI656100	0.159	0.185	0.010	0.028	6.164	15.100
PI533955	0.161	0.188	0.015	0.028	9.070	14.978
PI576376	0.192	0.205	0.031	0.023	16.249	11.055
PI576390	0.159	0.167	0.010	0.018	6.072	11.010
PI597966	0.196	0.173	0.039	0.042	19.865	24.355
PI533948	0.161	0.171	0.021	0.033	12.749	19.189
PI595743	0.164	0.192	0.023	0.032	13.821	16.566
PI534167	0.157	0.189	0.014	0.011	8.909	5.896
PI656026	0.184	0.153	0.025	0.038	13.601	24.876
PI656064	0.155	0.172	0.021	0.047	13.472	27.118
PI656077	0.171	0.150	0.020	0.026	11.563	17.472
PI656043	0.163	0.158	0.022	0.020	13.210	12.447
PI656038	0.177	0.168	0.025	0.046	14.234	27.269
PI656079	0.182	0.158	0.047	0.015	25.916	9.263
PI655991	0.173	0.160	0.027	0.018	15.585	11.206
PI656001	0.164	0.186	0.030	0.032	18.495	17.229
PI533937	0.200	0.162	0.013	0.021	6.345	12.758
PI533800	0.162	0.175	0.022	0.032	13.534	18.491
PI576333	0.170	0.184	0.023	0.035	13.575	18.936
PI656080	0.172	0.198	0.037	0.039	21.802	19.556
PI651492	0.157	0.168	0.021	0.031	13.216	18.384
PI576387	0.156	0.155	0.016	0.027	9.939	17.290
PI533876	0.169	0.157	0.031	0.033	18.619	20.883
PI656035	0.165	0.175	0.016	0.037	9.470	21.119
PI597976	0.166	0.157	0.023	0.049	13.608	30.891
PI656065	0.171	0.153	0.030	0.041	17.818	26.909
PI595745	0.173	0.161	0.026	0.016	14.811	10.033
PI533910	0.191	0.173	0.034	0.025	17.550	14.754
PI534097	0.161	0.160	0.019	0.037	11.726	22.902
PI533760	0.163	0.190	0.014	0.043	8.811	22.714
PI655979	0.169	0.171	0.015	0.019	8.915	10.919
PI595741	0.168	0.180	0.017	0.019	9.928	10.616
PI534099	0.157	0.169	0.023	0.020	14.671	11.830
PI656012	0.165	0.159	0.022	0.015	13.428	9.272
PI533976	0.186	0.185	0.025	0.024	13.434	12.896
PI613536	0.172	0.193	0.019	0.022	11.014	11.394
PI533833	0.161	0.153	0.023	0.037	13.958	24.233
PI533962	0.207	0.169	0.013	0.042	6.447	24.905
PI534075	0.188	0.175	0.030	0.036	15.845	20.535
PI533838	0.161	0.155	0.010	0.012	5.928	7.915
PI534101	0.169	0.171	0.022	0.023	13.055	13.738
PI576364	0.176	0.178	0.027	0.030	15.277	17.053
PI533964	0.163	0.160	0.012	0.013	7.347	8.392
PI533750	0.180	0.187	0.019	0.029	10.724	15.296
PI597965	0.165	0.173	0.012	0.029	7.029	16.976
PI533752	0.164	0.170	0.013	0.020	8.122	11.860

PI597968	0.166	0.165	0.013	0.024	7.848	14.421
PI655981	0.161	0.155	0.008	0.022	5.242	14.141
PI656034	0.189	0.175	0.031	0.033	16.537	18.934
PI48770	0.206	0.189	0.029	0.033	14.140	17.425
PI656018	0.166	0.161	0.013	0.009	7.581	5.798
PI656027	0.189	0.196	0.022	0.033	11.806	16.614
PI576375	0.188	0.177	0.027	0.028	14.496	15.774
PI656013	0.162	0.163	0.020	0.016	12.617	9.806
PI656086	0.167	0.169	0.024	0.024	14.218	14.040
PI152651	0.208	0.164	0.028	0.023	13.477	13.871
PI656050	0.168	0.169	0.016	0.016	9.671	9.560
PI656090	0.170	0.160	0.019	0.012	11.497	7.625
PI595744	0.170	0.169	0.032	0.029	18.815	17.004
PI533997	0.184	0.161	0.022	0.015	12.010	9.044
PI651496	0.162	0.167	0.017	0.014	10.319	8.521
PI534123	0.173	0.170	0.017	0.021	10.077	12.085
PI534139	0.170	0.180	0.017	0.021	10.053	11.644
PI655987	0.172	0.165	0.019	0.022	10.765	13.651
PI576359	0.177	0.166	0.017	0.022	9.594	13.134
PI656019	0.171	0.179	0.018	0.021	10.608	11.741
PI533980	0.174	0.157	0.026	0.023	15.128	14.563
PI585295	0.173	0.168	0.024	0.019	14.013	10.993
PI533762	0.178	0.170	0.024	0.022	13.678	13.122
PI533882	0.177	0.165	0.027	0.019	15.029	11.776
PI597980	0.174	0.182	0.017	0.026	9.850	14.274
PI656053	0.170	0.183	0.018	0.031	10.628	16.802
PI656030	0.174	0.178	0.022	0.028	12.814	15.584
PI533911	0.180	0.185	0.021	0.026	11.901	14.012
PI597973	0.168	0.180	0.017	0.019	9.834	10.479
PI656004	0.186	0.172	0.027	0.031	14.539	17.810
PI656109	0.173	0.175	0.022	0.025	12.950	14.102
PI533987	0.177	0.172	0.024	0.027	13.659	15.946
PI576426	0.178	0.179	0.022	0.027	12.583	15.112
PI533924	0.173	0.179	0.019	0.022	11.124	12.350
PI552856	0.172	0.176	0.018	0.028	10.322	16.140
PI576393	0.168	0.173	0.016	0.026	9.530	15.167
PI576340	0.175	0.172	0.021	0.030	11.768	17.304
PI533869	0.178	0.173	0.017	0.021	9.666	11.860
PI534163	0.172	0.169	0.016	0.026	9.261	15.422
PI595740	0.171	0.185	0.020	0.027	11.725	14.424
PI656096	0.185	0.181	0.019	0.028	10.395	15.672
PI534127	0.176	0.189	0.013	0.023	7.398	12.406
PI656066	0.171	0.171	0.020	0.021	11.399	12.437
PI576399	0.165	0.185	0.013	0.028	8.098	15.221
PI576345	0.165	0.178	0.013	0.018	8.185	9.917
PI655999	0.172	0.180	0.020	0.026	11.530	14.517
PI656083	0.174	0.180	0.015	0.030	8.415	16.524
PI656022	0.176	0.172	0.018	0.019	10.006	11.186

PI576348	0.168	0.163	0.014	0.011	8.607	6.977
PI656032	0.172	0.180	0.024	0.025	13.692	13.857
PI533902	0.174	0.172	0.028	0.033	15.989	19.101
PI534112	0.189	0.167	0.025	0.024	13.449	14.312
PI656058	0.183	0.180	0.030	0.031	16.404	17.221
PI533842	0.163	0.176	0.017	0.027	10.641	15.506
PI533996	0.182	0.201	0.030	0.038	16.263	19.044
PI576425	0.185	0.179	0.022	0.022	12.049	12.384
PI656114	0.183	0.166	0.027	0.028	14.591	16.981
PI576373	0.185	0.169	0.026	0.025	14.110	14.941
PI656087	0.168	0.184	0.023	0.029	13.374	15.734
PI595739	0.177	0.167	0.025	0.021	14.160	12.577
PI534054	0.174	0.194	0.025	0.035	14.154	18.296
PI576352	0.173	0.156	0.036	0.033	20.843	21.016
PI656016	0.184	0.165	0.029	0.013	15.992	7.629
PI533965	0.191	0.187	0.028	0.026	14.680	13.943
PI656082	0.180	0.182	0.026	0.033	14.244	17.863
PI564164	0.154	0.174	0.027	0.027	17.283	15.707
PI533871	0.201	0.191	0.009	0.033	4.551	17.464
PI656095	0.183	0.176	0.026	0.020	14.192	11.272
PI656103	0.185	0.168	0.027	0.038	14.582	22.685
PI34911	0.173	0.174	0.020	0.032	11.733	18.476
PI655995	0.187	0.204	0.029	0.027	15.769	13.457
PI656104	0.183	0.177	0.031	0.032	16.799	18.344
PI533855	0.181	0.207	0.028	0.028	15.306	13.428
PI595702	0.190	0.160	0.030	0.017	15.676	10.801
PI533863	0.184	0.192	0.029	0.041	15.554	21.284
PI656048	0.179	0.187	0.025	0.030	13.911	15.871
PI533989	0.178	0.180	0.024	0.042	13.686	23.319
PI534124	0.178	0.191	0.027	0.034	15.059	17.518
PI656115	0.173	0.160	0.023	0.015	13.490	9.115
PI642992	0.164	0.161	0.016	0.031	9.794	19.378
PI656102	0.179	0.163	0.033	0.013	18.209	7.881
PI533986	0.174	0.179	0.025	0.029	14.099	16.256
PI533991	0.190	0.158	0.031	0.027	16.520	16.854
PI656003	0.171	0.163	0.034	0.037	19.715	23.007
PI655992	0.181	0.165	0.024	0.022	13.415	13.479
PI576422	0.196	0.176	0.027	0.040	13.791	22.926
PI533757	0.167	0.169	0.044	0.015	26.650	8.989
PI595714	0.184	0.199	0.039	0.019	21.358	9.451
PI655985	0.192	0.163	0.041	0.030	21.363	18.531
PI533759	0.202	0.204	0.020	0.025	9.907	11.972
PI655993	0.209	0.187	0.034	0.015	16.076	8.182
PI533841	0.197	0.156	0.030	0.029	15.139	18.340
PI576332	0.183	0.186	0.041	0.038	22.270	20.651
PI597972	0.175	0.180	0.033	0.042	18.679	23.097
PI656108	0.197	0.209	0.037	0.034	19.028	16.230
PI534115	0.183	0.165	0.037	0.028	20.217	16.640

PI576385	0.181	0.194	0.039	0.032	21.747	16.361
PI533877	0.195	0.178	0.033	0.017	16.861	9.807
PI629034	0.195	0.175	0.048	0.041	24.585	23.167
PI533866	0.206	0.198	0.034	0.017	16.440	8.663
PI656112	0.159	0.170	0.025	0.047	15.583	27.444
PI533967	0.189	0.173	0.055	0.041	29.071	23.355
PI533961	0.203	0.184	0.039	0.042	18.975	22.627
PI534088	0.171	0.203	0.033	0.005	19.487	2.516
PI656041	0.192	0.150	0.022	0.023	11.456	15.839
PI534021	0.174	0.204	0.038	0.052	21.761	25.459
PI576381	0.191	0.191	0.032	0.025	16.797	13.009
PI533785	0.201	0.177	0.036	0.030	17.821	17.212
PI655998	0.177	0.185	0.045	0.021	25.384	11.575
PI656116	0.190	0.184	0.036	0.024	18.707	12.821
PI656071	0.188	0.155	0.034	0.016	18.146	10.454
PI656055	0.168	0.191	0.031	0.046	18.230	24.317
PI656075	0.194	0.154	0.032	0.035	16.781	22.558
PI655975	0.190	0.196	0.044	0.022	23.092	11.401
PI655997	0.196	0.202	0.035	0.021	18.014	10.252
PI534145	0.169	0.178	0.020	0.032	12.002	17.952
PI534108	0.198	0.204	0.019	0.031	9.650	15.349
PI548797	0.186	0.149	0.045	0.047	24.228	32.129
PI656078	0.190	0.203	0.044	0.030	23.384	14.780
PI656094	0.185	0.190	0.046	0.030	24.660	15.667
PI533776	0.167	0.192	0.036	0.040	21.735	20.798
PI641874	0.182	0.171	0.025	0.064	13.972	37.719
PI534155	0.209	0.187	0.023	0.031	10.792	16.763
PI533972	0.178	0.200	0.026	0.034	14.703	17.085
PI533755	0.191	0.187	0.027	0.026	14.277	13.696
PI533794	0.181	0.181	0.033	0.032	18.527	17.607
PI534114	0.180	0.181	0.027	0.035	14.727	19.126
PI534037	0.161	0.204	0.009	0.010	5.523	4.697
PI533766	0.164	0.198	0.027	0.027	16.761	13.442
PI534104	0.186	0.180	0.024	0.033	13.080	18.412
PI656052	0.176	0.162	0.026	0.016	15.025	10.129
PI533957	0.182	0.191	0.025	0.028	13.717	14.873
PI534157	0.170	0.193	0.019	0.037	11.340	19.293
PI595699	0.173	0.182	0.020	0.022	11.708	12.089
PI656068	0.186	0.183	0.026	0.029	14.141	15.656
PI656049	0.187	0.193	0.026	0.029	13.995	15.279
PI576437	0.176	0.180	0.021	0.024	11.985	13.470
PI542718	0.190	0.179	0.029	0.036	15.018	20.044
PI655973	0.203	0.177	0.030	0.022	14.560	12.224
PI561472	0.198	0.188	0.035	0.028	17.790	15.008
PI656046	0.205	0.189	0.025	0.037	12.232	19.810
PI656056	0.179	0.169	0.023	0.017	12.975	9.959
PI656025	0.183	0.176	0.029	0.032	15.783	17.937
PI534116	0.199	0.165	0.030	0.015	15.162	8.805

PI656093	0.173	0.199	0.029	0.034	16.723	16.892
PI656024	0.178	0.193	0.022	0.024	12.284	12.196
PI576428	0.160	0.184	0.017	0.037	10.360	19.938
PI656047	0.189	0.172	0.033	0.021	17.508	12.410
PI597946	0.169	0.166	0.022	0.029	12.847	17.204
PI656036	0.159	0.192	0.013	0.028	7.969	14.513
PI656101	0.188	0.175	0.034	0.019	18.226	10.729
PI534117	0.169	0.195	0.021	0.024	12.242	12.126
PI653616	0.189	0.183	0.025	0.029	12.964	15.884
PI534144	0.199	0.208	0.021	0.034	10.443	16.371
PI656059	0.189	0.173	0.031	0.026	16.135	15.178
PI655976	0.180	0.181	0.025	0.019	14.146	10.696
PI656000	0.171	0.179	0.015	0.026	8.565	14.261
PI656106	0.168	0.186	0.019	0.022	11.217	11.771
PI533970	0.171	0.173	0.014	0.027	8.474	15.388
PI576347	0.168	0.177	0.016	0.025	9.445	14.348
PI597960	0.184	0.182	0.023	0.024	12.636	13.017
PI533852	0.177	0.175	0.022	0.023	12.582	13.384
PI656017	0.183	0.185	0.022	0.025	11.880	13.527
PI533758	0.176	0.192	0.024	0.026	13.532	13.299
PI655971	0.181	0.179	0.024	0.022	13.373	12.113
PI533788	0.171	0.182	0.021	0.028	12.594	15.176
PI597971	0.159	0.182	0.005	0.027	2.878	14.996
PI533985	0.165	0.165	0.018	0.021	10.649	12.784
PI533769	0.169	0.184	0.015	0.026	9.195	13.931
PI629059	0.172	0.178	0.015	0.023	8.813	12.857
PI534138	0.173	0.172	0.023	0.022	13.427	12.570
PI534096	0.180	0.185	0.021	0.025	11.854	13.351
PI597961	0.183	0.178	0.024	0.023	13.012	12.918
PI656076	0.179	0.196	0.023	0.020	13.067	10.407
PI534009	0.186	0.170	0.028	0.021	14.910	12.228
PI656051	0.168	0.171	0.014	0.022	8.416	12.645
PI655972	0.184	0.177	0.025	0.028	13.556	15.660
PI655986	0.182	0.179	0.023	0.025	12.586	13.999
PI601816	0.172	0.190	0.021	0.024	12.045	12.731
PI655988	0.168	0.183	0.022	0.025	13.213	13.802
PI533938	0.182	0.184	0.023	0.026	12.656	14.146
PI533761	0.170	0.173	0.022	0.020	12.909	11.498
PI598069	0.175	0.173	0.020	0.024	11.468	13.972
PI576391	0.177	0.181	0.022	0.023	12.680	12.753
PI534079	0.172	0.185	0.024	0.026	13.832	14.140
PI561071	0.180	0.172	0.024	0.024	13.296	13.951
PI533979	0.180	0.177	0.028	0.021	15.384	12.062
PI534070	0.178	0.174	0.023	0.022	12.938	12.664
PI656037	0.171	0.177	0.022	0.029	12.917	16.502
PI576435	0.175	0.181	0.023	0.029	12.904	16.092
PI533940	0.178	0.189	0.019	0.026	10.752	13.937
PI656028	0.167	0.176	0.021	0.021	12.381	11.953

PI656031	0.179	0.184	0.022	0.025	12.323	13.607
PI656044	0.176	0.168	0.023	0.015	12.958	8.902
PI597945	0.172	0.195	0.017	0.032	10.012	16.467
PI656074	0.171	0.176	0.015	0.021	8.958	11.993
PI656033	0.201	0.176	0.026	0.031	13.092	17.714
PI595720	0.200	0.176	0.025	0.026	12.566	14.614
PI533814	0.193	0.175	0.027	0.024	13.866	14.001
PI534092	0.192	0.184	0.028	0.030	14.436	16.398
PI597949	0.182	0.194	0.028	0.030	15.429	15.658
PI656070	0.207	0.188	0.028	0.028	13.654	14.846
PI656085	0.173	0.185	0.027	0.030	15.734	16.037
PI533878	0.175	0.163	0.022	0.014	12.715	8.337
PI656120	0.179	0.182	0.024	0.026	13.211	14.194
PI655978	0.184	0.185	0.023	0.031	12.404	16.566
PI533824	0.193	0.194	0.029	0.032	15.234	16.418
PI656072	0.148	0.178	0.031	0.025	20.826	14.148
PI576380	0.177	0.180	0.026	0.027	14.676	14.815
PI656092	0.175	0.196	0.025	0.025	14.477	12.881
PI597964	0.186	0.181	0.033	0.028	17.755	15.289
PI534135	0.189	0.195	0.026	0.032	13.917	16.345
PI533822	0.160	0.180	0.014	0.028	8.966	15.703
PI656069	0.178	0.198	0.026	0.027	14.621	13.785
PI534133	0.168	0.189	0.023	0.031	13.606	16.403
PI533943	0.203	0.175	0.031	0.028	15.374	16.245
PI533810	0.179	0.184	0.023	0.028	13.071	15.115
PI534148	0.180	0.192	0.024	0.033	13.158	17.301
PI656042	0.197	0.186	0.019	0.030	9.659	15.968
PI564165	0.193	0.175	0.026	0.026	13.538	14.624
PI540816	0.207	0.209	0.031	0.023	14.932	10.987
PI656091	0.199	0.159	0.029	0.025	14.671	15.766
PI576366	0.192	0.174	0.026	0.025	13.442	14.208
PI533799	0.188	0.201	0.025	0.032	13.320	16.007
PI656121	0.176	0.196	0.011	0.034	6.118	17.478
PI656014	0.177	0.175	0.023	0.025	12.753	14.144
PI533939	0.184	0.173	0.028	0.029	15.148	16.784
PI534137	0.181	0.183	0.026	0.032	14.319	17.189
PI533845	0.170	0.178	0.023	0.026	13.844	14.846
PI656081	0.183	0.173	0.028	0.022	15.155	12.478
PI533998	0.194	0.184	0.035	0.028	18.101	15.137
PI533754	0.186	0.195	0.030	0.032	16.218	16.334
PI552861	0.184	0.182	0.027	0.023	14.445	12.738
PI533843	0.186	0.183	0.032	0.025	16.975	13.569
PI597982	0.166	0.184	0.012	0.030	7.119	16.250
PI534128	0.189	0.183	0.025	0.027	13.187	14.556
PI655990	0.190	0.193	0.032	0.031	17.051	15.819
PI656105	0.186	0.182	0.027	0.025	14.466	13.455
PI595718	0.182	0.201	0.025	0.032	13.713	15.747
PI656117	0.189	0.194	0.024	0.028	12.830	14.362

PI597967	0.191	0.199	0.032	0.024	16.953	12.061
PI656063	0.189	0.191	0.025	0.032	13.081	16.565
PI597957	0.180	0.173	0.031	0.019	17.351	11.110
PI533856	0.189	0.190	0.025	0.028	13.449	14.909
PI576349	0.192	0.191	0.033	0.033	17.157	17.176
PI655974	0.188	0.165	0.029	0.038	15.182	23.303
PI656111	0.190	0.181	0.026	0.023	13.810	12.840
PI656089	0.185	0.189	0.028	0.022	14.937	11.770
PI534063	0.187	0.184	0.026	0.025	14.058	13.820
PI561073	0.194	0.170	0.026	0.017	13.354	9.991
PI641849	0.185	0.183	0.031	0.031	16.838	17.172
PI533956	0.193	0.171	0.024	0.019	12.587	11.167
PI533807	0.190	0.183	0.029	0.028	15.147	15.133
PI656119	0.175	0.187	0.019	0.030	10.997	15.890
PI576130	0.170	0.186	0.019	0.028	11.079	15.308
PI655970	0.188	0.184	0.025	0.042	13.451	22.593
PI576339	0.197	0.175	0.028	0.019	14.404	10.795
PI576350	0.180	0.195	0.020	0.032	10.832	16.390
PI597950	0.185	0.192	0.018	0.035	9.895	18.146
PI598070	0.186	0.183	0.029	0.026	15.804	14.042
PI656062	0.173	0.190	0.015	0.025	8.940	13.141
PI656015	0.192	0.191	0.023	0.029	11.707	15.000
PI597951	0.201	0.178	0.033	0.020	16.458	11.254
PI656011	0.189	0.202	0.024	0.023	12.797	11.581
PI656023	0.182	0.186	0.048	0.026	26.369	13.713
PI655980	0.199	0.197	0.027	0.030	13.511	15.077
PI609456	0.195	0.194	0.029	0.029	14.781	14.929
PI576418	0.185	0.197	0.021	0.032	11.312	16.448
PI656107	0.197	0.188	0.030	0.024	15.095	12.983
PI576394	0.205	0.194	0.032	0.021	15.539	10.881
PI533936	0.205	0.191	0.026	0.021	12.918	11.025
PI656118	0.183	0.189	0.023	0.030	12.436	15.663
PI576386	0.204	0.183	0.028	0.035	13.595	19.030
PI276837	0.177	0.185	0.018	0.028	9.903	15.184
PI655994	0.189	0.191	0.025	0.029	13.424	15.107

Dissecting the genetic control of natural variation in sorghum photosynthetic response to drought stress

Diego Ortiz, Maria G. Salas-Fernandez

Supplementary Table S2. Phenotypic correlations between photosynthesis and chlorophyll fluorescence traits based on BLUPs in control period.

	Correlation (r)							
Traits	A	E	g_s	F_v/F_m	F_v'/F_m'	φ_{PSII}	qP	$A:E$
A	-							
E	0.77***	-						
g_s	0.82***	0.9***	-					
F_v/F_m	0.22***	0.09	0.13*	-				
F_v'/F_m'	0.68***	0.51***	0.6***	0.42***	-			
φ_{PSII}	0.83***	0.59***	0.71***	0.28***	0.77***	-		
qP	0.61***	0.42***	0.52***	0.04	0.3***	0.8***	-	
$A:E$	-0.05	-0.66***	-0.45***	0.11	-0.02	0.04	0.05	-

A= photosynthesis (μmol CO_2 m^{-2} s^{-1}) ; E= transpiration rate (mmol H_20 m^{-2} s^{-1}), g_s= stomatal conductance (mol H_20 m^{-2} s^{-1}), F_v/F_m = maximum quantum yield of PSII, Φ_{PSII}=effective quantum yield of PSII; F_v'/F_m'=efficiency of energy captured by open PSII reaction centers , qP=photo-chemical quenching or fraction of PSII reaction centers that are open, and A:E= ratio A:E; *Significant at $P < 0.05$;** Significant at $P < 0.01$;*** Significant at $P < 0.001$

Supplementary Table S3. Phenotypic correlations between photosynthetic and chlorophyll fluorescence traits based on BLUPs in drought period.

	Correlation (r)							
Traits	A	E	g_s	F_v/F_m	F_v'/F_m'	φ_{PSII}	qP	$A:E$
A	-							
E	0.98***	-						
g_s	0.98***	0.99***	-					
F_v/F_m	0.4***	0.39***	0.4***	-				
F_v'/F_m'	0.86***	0.86***	0.86***	0.49***	-			
φ_{PSII}	0.97***	0.95***	0.96***	0.41***	0.87***	-		
qP	0.88***	0.85***	0.84***	0.25***	0.61***	0.91***	-	
$A:E$	0.01	-0.17**	-0.12*	-0.02	-0.09	-0.02	0.07	-

A= photosynthesis (μmol CO_2 m^{-2} s^{-1}) ; E= transpiration rate (mmol H_20 m^{-2} s^{-1}), g_s= stomatal conductance (mol H_20 m^{-2} s^{-1}), F_v/F_m = maximum quantum yield of PSII, Φ_{PSII}=effective quantum yield of PSII; F_v'/F_m'=efficiency of energy captured by open PSII reaction centers , qP=photo-chemical quenching or fraction of PSII reaction centers that are open, and A:E= ratio A:E; *Significant at $P < 0.05$;** Significant at $P < 0.01$;*** Significant at $P < 0.001$

Supplementary Table S4. Phenotypic correlations between photosynthetic and chlorophyll fluorescence traits based on BLUPs in recovery period.

	Correlation (r)							
Traits	*A*	*E*	g_s	F_v/F_m	F_v'/F_m'	φ_{PSII}	*qP*	*A:E*
A	-							
E	0.85***	-						
g_s	0.91***	0.95***	-					
F_v/F_m	0.32***	0.31***	0.33***	-				
F_v'/F_m'	0.75***	0.69***	0.74***	0.33***	-			
φ_{PSII}	0.88***	0.74***	0.8***	0.27***	0.8***	-		
qP	0.59***	0.42***	0.48***	0.04	0.19***	0.73***	-	
A:E	-0.05	-0.52***	-0.35***	-0.03	-0.15**	-0.02	0.13*	-

A= photosynthesis (μmol CO_2 m^{-2} s^{-1}) ; *E*= transpiration rate (mmol H_20 m^{-2} s^{-1}), g_s= stomatal conductance (mol H_20 m^{-2} s^{-1}), F_v/F_m = maximum quantum yield of PSII, Φ_{PSII}=effective quantum yield of PSII; F_v'/F_m'=efficiency of energy captured by open PSII reaction centers , *qP*=photo-chemical quenching or fraction of PSII reaction centers that are open, and A:E= ratio A:E; *Significant at $P < 0.05$;** Significant at $P < 0.01$;*** Significant at $P < 0.001$

Supplementary Table S5. Analysis of variance of photosynthesis and chlorophyll fluorescence traits in control, drought and recovery periods.

	***A* control**		***A* drought**		***A* recovery**	
Fixed effects	F Value	Pr > F	F Value	Pr > F	F Value	Pr > F
Day	9.17	0.0001	93.38	<.0001	502.08	<.0001
machine	22.28	<.0001	22.43	<.0001	32.87	<.0001
tleaf			457.08	<.0001	8.59	0.0035
Random effects		LR p-value		LR p-value		LR p-value
set		0.015		2.03E-04		0.014
Rep(set)		1.53E-05		2.96E-11		0.061
Geno		2.46E-13		4.26E-12		3.30E-12

	***E* control**		***E* drought**		***E* recovery**	
Fixed effects	F Value	Pr > F	F Value	Pr > F	F Value	Pr > F
Day	1.56	0.21	101.87	<.0001	281.26	<.0001
machine	60.49	<.0001	29.90	<.0001	18.28	<.0001
tleaf			249.81	<.0001	14.79	0.0001
Random effects		LR p-value		LR p-value		LR p-value
set		0.480		0.001		0.013
Rep(set)		3.30E-20		1.61E-09		0.038
Geno		1.38E-09		4.94E-11		4.49E-09

	**g_s control**		**g_s drought**		**g_s recovery**	
Fixed effects	F Value	Pr > F	F Value	Pr > F	F Value	Pr > F
Day	0.70	0.4961	112.72	<.0001	400.70	<.0001
machine	19.22	<.0001	24.61	<.0001	24.07	<.0001
tleaf			486.98	<.0001	1.10	0.2947
Random effects		LR p-value		LR p-value		LR p-value
set		0.078		5.41E-05		0.038
Rep(set)		1.13E-08		3.13E-09		0.094
Geno		1.39E-12		1.29E-14		6.38E-11

	F_v/F_m control		F_v/F_m drought		F_v/F_m recovery	
Fixed effects	F Value	Pr > F	F Value	Pr > F	F Value	Pr > F
Day	6.22	0.0021	129.81	<.0001	474.13	<.0001
Random effects		LR p-value		LR p-value		LR p-value
set		3.08E-07		2.52E-04		0.403
Rep(set)		0.655		5.44E-02		0.004
Geno		5.10E-06		1.13E-04		4.87E-05

	F_v'/F_m' control		F_v'/F_m' drought		F_v'/F_m' recovery	
Fixed effects	F Value	Pr > F	F Value	Pr > F	F Value	Pr > F
Day	1.46	0.2332	148.89	<.0001	211.03	<.0001
machine	7.76	0.0005	27.79	<.0001	0.19	0.8264
tleaf			349.36	<.0001	15.56	<.0001
Random effects		LR p-value		LR p-value		LR p-value
set		0.046		6.01E-05		0.480
Rep(set)		8.67E-11		5.41E-05		0.004
Geno		1.96E-15		1.15E-16		1.31E-15

	φ_{PSII} control		φ_{PSII} drought		φ_{PSII} recovery	
Fixed effects	F Value	Pr > F	F Value	Pr > F	F Value	Pr > F
Day	6.09	0.0024	121.84	<.0001	399.53	<.0001
machine	30.09	<.0001	67.61	<.0001	14.87	<.0001
tleaf			372.46	<.0001	25.75	<.0001
Random effects		LR p-value		LR p-value		LR p-value
set		0.046		2.33E-05		0.078
Rep(set)		2.38E-07		2.59E-06		0.403
Geno		1.38E-09		1.12E-11		8.32E-09

	qP control		*qP* drought		*qP* recovery	
Fixed effects	F Value	Pr > F	F Value	Pr > F	F Value	Pr > F
Day	5.29	0.0053	79.09	<.0001	289.81	<.0001
machine	44.54	<.0001	84.45	<.0001	34.61	<.0001
tleaf			279.64	<.0001	11.40	0.0008
Random effects		LR p-value		LR p-value		LR p-value
set		0.034		9.17E-05		0.074
Rep(set)		0.002		3.55E-05		0.021
Geno		3.19E-06		9.55E-06		1.48E-04

	***A:E* control**		***A:E* drought**		***A:E* recovery**	
Fixed effects	F Value	Pr > F	F Value	Pr > F	F Value	Pr > F
Day	0.50	0.6076	6.14	0.0023	0.29	0.5909
machine	61.59	<.0001	15.14	<.0001	13.67	<.0001
tleaf			123.45	<.0001	7.48	0.0063
Random effects		LR p-value		LR p-value		LR p-value
set		1.000		0.006		0.032
Rep(set)		3.60E-32		1.65E-07		2.33E-16
Geno		2.73E-05		0.294		0.273

Supplementary Table S6. Summary of GWAs results for gas exchange and chlorophyll fluorescence traits in control, drought and recovery periods, and derived variables cumulative response, and ratio drought-recovery.

Trait	Treatment	Chr.	Marker	p value	q value	R^2	LD region #
A	recovery	1	*S1_2949673*	1.67E-05	0.131704	0.06201	1_1
A	recovery	1	*S1_8612970*	2.91E-05	0.131704	0.07238	1_10
gs	recovery	1	*S1_8612970*	1.72E-05	0.085417	0.08198	1_10
A	recovery	1	*S1_8621860*	5.64E-05	0.131704	0.06206	1_11
A	recovery	1	*S1_8777957*	3.02E-05	0.131704	0.07877	1_12
E	recovery	1	*S1_8777957*	1.68E-05	0.122096	0.08627	1_12
gs	recovery	1	*S1_8777957*	9.63E-06	0.085417	0.08959	1_12
Fv'/Fm'	cumulative response	1	*S1_8934452*	4.97E-06	0.184332	0.09475	1_13
qP	control	1	*S1_9132031*	8.75E-06	0.176533	0.08258	1_14
A	recovery	1	*S1_10003241*	9.56E-06	0.131704	0.08814	1_15
gs	recovery	1	*S1_10003241*	9.41E-06	0.085417	0.08975	1_15
A	recovery	1	*S1_10025602*	8.94E-06	0.131704	0.10366	1_15
E	recovery	1	*S1_10025602*	6.15E-06	0.122096	0.11254	1_15
gs	recovery	1	*S1_10025602*	2.57E-06	0.085417	0.12006	1_15
Fv'/Fm'	recovery	1	*S1_10025602*	4.22E-07	0.044573	0.12806	1_15
φ_{PSII}	recovery	1	*S1_10025602*	1.42E-05	0.117732	0.10414	1_15
A	recovery	1	*S1_10172070*	3.03E-05	0.131704	0.07289	1_15
A	recovery	1	*S1_11229843*	1.10E-05	0.131704	0.07846	1_15
gs	recovery	1	*S1_11229843*	4.12E-05	0.086534	0.06943	1_15
Fv'/Fm'	recovery	1	*S1_11229843*	3.63E-06	0.058684	0.08399	1_15
A	recovery	1	*S1_11229844*	1.10E-05	0.131704	0.07846	1_15
gs	recovery	1	*S1_11229844*	4.12E-05	0.086534	0.06943	1_15
Fv'/Fm'	recovery	1	*S1_11229844*	3.63E-06	0.058684	0.08399	1_15
gs	recovery	1	*S1_17880348*	2.78E-05	0.085417	0.06394	1_16
qP	control	1	*S1_18981548*	1.08E-05	0.176533	0.06616	1_17
Fv'/Fm'	recovery	1	*S1_19558512*	5.15E-06	0.058684	0.07089	1_18
φ_{PSII}	recovery	1	*S1_19558512*	1.13E-05	0.117732	0.07165	1_18
A	recovery	1	*S1_26706152*	5.59E-05	0.131704	0.06047	1_19
Fv'/Fm'	recovery	1	*S1_26706152*	5.26E-06	0.058684	0.08053	1_19
A	control	1	*S1_3299477*	5.62E-07	0.07347	0.09042	1_2
A	recovery	1	*S1_48981201*	3.57E-05	0.131704	0.07915	1_20
φ_{PSII}	recovery	1	*S1_48981201*	5.33E-06	0.117732	0.09335	1_20
Fv'/Fm'	cumulative response	1	*S1_49906006*	4.18E-06	0.184332	0.08843	1_21
A	recovery	1	*S1_51100106*	3.27E-05	0.131704	0.0554	1_22
φ_{PSII}	recovery	1	*S1_51100106*	1.95E-05	0.126506	0.05887	1_22
A	recovery	1	*S1_51100116*	3.27E-05	0.131704	0.0554	1_22
φ_{PSII}	recovery	1	*S1_51100116*	1.95E-05	0.126506	0.05887	1_22
A	recovery	1	*S1_51100176*	3.01E-05	0.131704	0.07037	1_22
φ_{PSII}	recovery	1	*S1_51100176*	9.60E-06	0.117732	0.07636	1_22
A	recovery	1	*S1_51100209*	3.01E-05	0.131704	0.07037	1_22

φ_{PSII}	recovery	1	*S1_51100209*	9.60E-06	0.117732	0.07636	1_22
A	recovery	1	*S1_51420743*	3.01E-05	0.131704	0.06663	1_23
A	recovery	1	*S1_61726216*	5.40E-05	0.131704	0.06515	1_24
A	recovery	1	*S1_61726220*	5.40E-05	0.131704	0.06515	1_24
A	recovery	1	*S1_61729859*	5.34E-05	0.131704	0.05977	1_25
gs	recovery	1	*S1_5689384*	3.94E-05	0.086534	0.05875	1_3
A	recovery	1	*S1_7320011*	3.34E-05	0.131704	0.06485	1_4
gs	recovery	1	*S1_7320011*	3.74E-05	0.086534	0.06117	1_4
gs	recovery	1	*S1_7320028*	3.05E-05	0.085417	0.06453	1_5
gs	recovery	1	*S1_7320032*	3.05E-05	0.085417	0.06453	1_5
A	recovery	1	*S1_7356148*	5.59E-05	0.131704	0.05153	1_6
A	recovery	1	*S1_7370295*	4.86E-05	0.131704	0.05488	1_6
A	recovery	1	*S1_7663160*	2.76E-05	0.131704	0.07558	1_6
E	recovery	1	*S1_7663160*	5.55E-06	0.122096	0.08768	1_6
gs	recovery	1	*S1_7663160*	6.16E-06	0.085417	0.08593	1_6
A	recovery	1	*S1_7691839*	4.15E-05	0.131704	0.06475	1_7
gs	recovery	1	*S1_7691839*	2.66E-05	0.085417	0.06922	1_7
A	recovery	1	*S1_8002459*	5.60E-05	0.131704	0.05301	1_8
gs	recovery	1	*S1_8002459*	3.01E-05	0.085417	0.05724	1_8
Fv'/Fm'	recovery	1	*S1_8002459*	8.50E-06	0.058684	0.06365	1_8
A	recovery	1	*S1_8152122*	2.00E-05	0.131704	0.07696	1_9
gs	recovery	1	*S1_8152122*	3.14E-05	0.085417	0.0698	1_9
A:E	recovery	10	*S10_6278477*	1.73E-06	0.115983	0.08039	10_1
A:E	recovery	10	*S10_6278507*	1.73E-06	0.115983	0.08039	10_1
qP	control	10	*S10_9792811*	1.30E-05	0.176533	0.08948	10_2
A	recovery	10	*S10_16057480*	5.15E-05	0.131704	0.0513	10_3
A	recovery	10	*S10_16803467*	1.95E-05	0.131704	0.07025	10_3
gs	recovery	10	*S10_16803467*	4.03E-05	0.086534	0.06333	10_3
A	recovery	10	*S10_17059423*	5.20E-05	0.131704	0.0697	10_3
A	recovery	10	*S10_17059425*	5.20E-05	0.131704	0.0697	10_3
A	recovery	10	*S10_17059456*	1.87E-05	0.131704	0.07914	10_3
A	recovery	10	*S10_17060480*	4.49E-05	0.131704	0.05291	10_3
E	recovery	10	*S10_17216310*	1.80E-05	0.122096	0.0603	10_4
gs	recovery	10	*S10_17216310*	3.24E-05	0.085417	0.05734	10_4
E	recovery	10	*S10_17216319*	1.80E-05	0.122096	0.0603	10_4
gs	recovery	10	*S10_17216319*	3.24E-05	0.085417	0.05734	10_4
gs	recovery	10	*S10_17424736*	3.17E-05	0.085417	0.05467	10_4
A	recovery	10	*S10_17670660*	1.28E-05	0.131704	0.06599	10_4
gs	recovery	10	*S10_17670660*	2.38E-05	0.085417	0.06176	10_4
A	recovery	10	*S10_17919861*	1.14E-05	0.131704	0.06455	10_4
E	recovery	2	*S2_5679675*	1.32E-06	0.050601	0.09104	2_1
gs	recovery	2	*S2_5679675*	6.20E-06	0.085417	0.07823	2_1
E	recovery	2	*S2_5680569*	1.61E-05	0.122096	0.06266	2_1
gs	recovery	2	*S2_5680569*	2.89E-05	0.085417	0.05949	2_1
E	cumulative response	2	*S2_5742374*	5.32E-06	0.118576	0.10309	2_2
E	recovery	2	*S2_58963955*	2.91E-07	0.033593	0.11474	2_3

gs	recovery	2	*S2_58963955*	6.04E-08	0.005918	0.12832	2_3
E	cumulative response	2	*S2_58963955*	4.17E-06	0.116216	0.09492	2_3
Fv'/Fm'	recovery	2	*S2_58963955*	2.61E-06	0.058684	0.09408	2_3
gs	recovery	2	*S2_67521242*	3.31E-05	0.085417	0.05647	2_4
E	recovery	2	*S2_70749331*	1.21E-05	0.122096	0.06071	2_5
gs	recovery	2	*S2_70749331*	1.78E-05	0.085417	0.05968	2_5
gs	recovery	3	*S3_56905719*	3.17E-05	0.085417	0.06959	3_1
A	recovery	4	*S4_3814427*	4.53E-05	0.131704	0.05741	4_1
φ_{PSII}	recovery	4	*S4_3814427*	1.59E-05	0.117732	0.06796	4_1
A	recovery	4	*S4_3823650*	3.93E-05	0.131704	0.06192	4_1
φ_{PSII}	recovery	4	*S4_3823650*	1.32E-05	0.117732	0.06944	4_1
A	recovery	4	*S4_3823654*	4.39E-05	0.131704	0.05822	4_1
φ_{PSII}	recovery	4	*S4_3823654*	1.60E-05	0.117732	0.06482	4_1
φ_{PSII}	recovery	4	*S4_3836151*	6.30E-06	0.117732	0.07512	4_2
Fv'/Fm'	recovery	4	*S4_3929400*	7.63E-06	0.058684	0.08497	4_2
φ_{PSII}	recovery	4	*S4_3929400*	1.98E-06	0.117732	0.10291	4_2
E	recovery	4	*S4_5723712*	1.79E-05	0.122096	0.06746	4_2
gs	recovery	4	*S4_5723712*	1.16E-05	0.085417	0.07175	4_2
φ_{PSII}	recovery	4	*S4_5723712*	1.43E-05	0.117732	0.07183	4_2
E	recovery	4	*S4_5723722*	1.79E-05	0.122096	0.06746	4_2
gs	recovery	4	*S4_5723722*	1.16E-05	0.085417	0.07175	4_2
φ_{PSII}	recovery	4	*S4_5723722*	1.43E-05	0.117732	0.07183	4_2
E	recovery	4	*S4_5723735*	1.79E-05	0.122096	0.06746	4_2
gs	recovery	4	*S4_5723735*	1.16E-05	0.085417	0.07175	4_2
φ_{PSII}	recovery	4	*S4_5723735*	1.43E-05	0.117732	0.07183	4_2
E	recovery	4	*S4_5723965*	1.24E-05	0.122096	0.06905	4_2
gs	recovery	4	*S4_5723965*	5.22E-06	0.085417	0.07679	4_2
φ_{PSII}	recovery	4	*S4_5723965*	5.97E-06	0.117732	0.08114	4_2
E	recovery	4	*S4_5723985*	1.24E-05	0.122096	0.06905	4_2
gs	recovery	4	*S4_5723985*	5.22E-06	0.085417	0.07679	4_2
φ_{PSII}	recovery	4	*S4_5723985*	5.97E-06	0.117732	0.08114	4_2
gs	recovery	4	*S4_46364030*	2.98E-05	0.085417	0.05609	4_3
gs	recovery	4	*S4_46364036*	2.98E-05	0.085417	0.05609	4_3
gs	recovery	4	*S4_46364048*	2.98E-05	0.085417	0.05609	4_3
gs	recovery	4	*S4_54374776*	2.02E-05	0.085417	0.08401	4_4
gs	recovery	4	*S4_54915096*	3.16E-05	0.085417	0.06467	4_5
A	cumulative response	4	*S4_61122741*	4.50E-07	0.049851	0.08955	4_6
E	drought	4	*S4_61122741*	2.77E-06	0.120964	0.0818	4_6
E	cumulative response	4	*S4_61122741*	3.62E-07	0.040417	0.09437	4_6
E	drought	4	*S4_61757012*	5.36E-06	0.155114	0.08562	4_7
E	drought	4	*S4_61776859*	1.36E-06	0.120964	0.1106	4_7
E	cumulative response	4	*S4_61776859*	3.46E-06	0.116216	0.10521	4_7
Fv'/Fm'	ratio DC	4	*S4_61776859*	9.36E-07	0.111491	0.10985	4_7
φ_{PSII}	ratio DC	4	*S4_61776859*	1.28E-06	0.156875	0.10768	4_7
qP	control	4	*S4_64314021*	1.23E-05	0.176533	0.07055	4_8

A	recovery	5	*S5_3355698*	8.91E-06	0.131704	0.07011	5_1
E	recovery	5	*S5_3355698*	5.96E-07	0.034354	0.08724	5_1
gs	recovery	5	*S5_3355698*	1.04E-07	0.005918	0.10177	5_1
Fv'/Fm'	recovery	5	*S5_9588341*	8.35E-06	0.058684	0.08134	5_2
Fv'/Fm'	recovery	5	*S5_9588371*	8.35E-06	0.058684	0.08134	5_2
Fv'/Fm'	recovery	5	*S5_9588375*	8.35E-06	0.058684	0.08134	5_2
Fv'/Fm'	recovery	5	*S5_9589605*	9.45E-06	0.058684	0.08401	5_2
Fv'/Fm'	recovery	5	*S5_9589610*	9.45E-06	0.058684	0.08401	5_2
Fv'/Fm'	recovery	5	*S5_9589630*	9.45E-06	0.058684	0.08401	5_2
E	drought	5	*S5_42764230*	3.13E-06	0.120964	0.07683	5_3
E	cumulative response	5	*S5_42764230*	3.09E-06	0.116216	0.07685	5_3
Fv'/Fm'	cumulative response	5	*S5_42764230*	4.98E-06	0.184332	0.07418	5_3
A	recovery	5	*S5_59228941*	3.61E-05	0.131704	0.06706	5_4
gs	recovery	5	*S5_59228941*	4.34E-06	0.085417	0.08296	5_4
A	recovery	5	*S5_59228968*	5.19E-05	0.131704	0.06817	5_4
gs	recovery	5	*S5_59228968*	1.25E-05	0.085417	0.078	5_4
E	recovery	5	*S5_62168822*	1.15E-05	0.122096	0.06563	5_5
gs	recovery	5	*S5_62168822*	1.41E-05	0.085417	0.06521	5_5
Fv'/Fm'	recovery	5	*S5_62168822*	5.31E-06	0.058684	0.07	5_5
gs	recovery	5	*S5_62168880*	2.89E-05	0.085417	0.06598	5_5
gs	recovery	5	*S5_62168893*	2.89E-05	0.085417	0.06598	5_5
qP	control	6	*S6_59577575*	7.73E-06	0.176533	0.07246	6_1
qP	control	6	*S6_59932272*	2.66E-06	0.176533	0.08758	6_2
qP	control	6	*S6_59980948*	1.22E-05	0.176533	0.07042	6_2
qP	control	6	*S6_60110274*	8.79E-06	0.176533	0.0705	6_3
qP	control	6	*S6_60110289*	8.79E-06	0.176533	0.0705	6_3
Fv'/Fm'	recovery	7	*S7_5256995*	5.34E-06	0.058684	0.08663	7_1
A	recovery	7	*S7_12865678*	2.22E-05	0.131704	0.06095	7_2
gs	recovery	7	*S7_12865678*	3.55E-05	0.086534	0.05693	7_2
A	recovery	7	*S7_13086524*	4.46E-05	0.131704	0.05343	7_2
gs	recovery	7	*S7_13086524*	3.26E-05	0.085417	0.05579	7_2
A	recovery	7	*S7_13086535*	3.70E-05	0.131704	0.05592	7_2
gs	recovery	7	*S7_13086535*	3.76E-05	0.086534	0.05575	7_2
A	recovery	7	*S7_13086537*	3.79E-05	0.131704	0.05583	7_2
gs	recovery	7	*S7_13086537*	3.84E-05	0.086534	0.05566	7_2
A	recovery	7	*S7_13086538*	3.65E-05	0.131704	0.05605	7_2
gs	recovery	7	*S7_13086538*	3.75E-05	0.086534	0.05578	7_2
gs	recovery	7	*S7_58584926*	2.50E-05	0.085417	0.06759	7_3
Fv'/Fm'	recovery	7	*S7_59050234*	7.72E-06	0.058684	0.08043	7_3
gs	recovery	8	*S8_881633*	3.12E-05	0.085417	0.08735	8_1
gs	recovery	8	*S8_881693*	3.97E-05	0.086534	0.05951	8_1
A	recovery	8	*S8_1292148*	2.16E-05	0.131704	0.08357	8_2
gs	recovery	8	*S8_4642875*	2.10E-05	0.085417	0.06761	8_3
E	recovery	8	*S8_5900864*	1.44E-05	0.122096	0.09581	8_4
gs	recovery	8	*S8_5949187*	3.31E-05	0.085417	0.05911	8_5
A	recovery	8	*S8_6112580*	3.83E-05	0.131704	0.06184	8_6
E	recovery	8	*S8_6112580*	2.03E-05	0.13028	0.06431	8_6

gs	recovery	8	*S8_6112580*	1.06E-05	0.085417	0.06992	8_6
gs	recovery	8	*S8_6112866*	2.42E-05	0.085417	0.07511	8_6
A	recovery	8	*S8_6269394*	1.07E-05	0.131704	0.08089	8_6
